# Creating order in the uncertainty of childbirth: A qualitative study of obstetric admission and triage practices in rural Malawi

**DOI:** 10.1371/journal.pgph.0006312

**Published:** 2026-10-26

**Authors:** Emily Bundy, Bianca Kandeya, Wanangwa Chimwaza, Claudia Hanson, Effie Chipeta, Helle Mölsted Alvesson

**Affiliations:** 1 Department of Global Public Health, Karolinska Institutet, Stockholm, Sweden; 2 Centre for Reproductive Health, Kamuzu University of Health Sciences, Blantyre, Malawi; PLOS: Public Library of Science, UNITED STATES OF AMERICA

## Abstract

Maternal and neonatal mortality continues to be a concern in sub-Saharan Africa. Although standardized obstetric triage improves clinical outcomes, little is known about how locally developed admission practices function in low-resource settings where national obstetric triage guidelines are absent. This study explored how hospital-based obstetric admission procedures in rural Malawi were experienced by maternity care providers, laboring women, and companions. We used qualitative methods including in-depth interviews with mothers and maternity care providers, focus group discussions with companions and maternity care providers, and observations of two labor wards. Study participants were chosen purposively to represent diverse birth outcomes and professionals. Data analysis followed reflexive thematic analysis, concentrating on obstetric admissions. Mary Douglas’ concepts of purity, danger, and risk informed interpretation during theme development. Obstetric admissions were organized through standardized admission forms that structured assessment and enabled providers to classify women according to visible danger signs and labor progression. Although the forms were not formal triage tools, they functioned as the primary mechanism for prioritizing care, organizing patient movement through maternity spaces, and managing high patient volumes. Shared recognition of danger signs among providers, women, and companions reinforced the legitimacy of risk-based decision making. At the same time, communication was selectively restricted, limiting patient and companion participation while reinforcing professional authority and hierarchical relationships. Standardized admission procedures created order by classifying obstetric risk and prioritizing clinical efficiency, but they also reinforced hierarchical relationships that limited communication and patient participation. Efforts to strengthen obstetric admissions should preserve the benefits of standardized risk assessment while incorporating more person-centered communication and companion engagement.

## Introduction

Despite global progress, maternal and neonatal mortality rates remain unacceptably high in sub-Saharan Africa, with current estimates at 545 maternal deaths per 100,000 live births and 28 neonatal deaths per 1,000 live births [1–3]. Most of these deaths occur during the perinatal period which is defined as the time between 22 weeks’ gestation and seven days postpartum, a critical window for intervention [1,4]. While still a novel practice in many global settings, the successful implementation of reliable obstetric triaging systems has positively impacted perinatal mortality in both low and high resourced settings [5–7]. Standardized risk assessment at admission to childbirth and the triage systems that follow promise greater efficiency and are well established in high-income countries. In contrast, many low-and middle-income countries (LMICs) lack comparable standardized approaches, as global guidance has not yet provided context specific recommendations for obstetric triage and previous implementation efforts have demonstrated limited success [8]. As a result, although formal triage has not been introduced, concepts originating in HICs continue to shape practice in LMICs, leading to a patchwork of locally developed approaches [5–7,9]. Obstetric triage considers the duality of the mother and fetus through fetal assessment, labor evaluation, and diagnostic testing for obstetric complications to prioritize care based on acuity [10,11]. Effective obstetric triage with well-defined admission criteria reduces patient waiting times, improves accuracy of diagnosis, increases the appropriateness of care plans, enables the standardization of initial assessment, and ultimately enhances patient satisfaction while also improving clinical outcomes [12,13]. However, perinatal outcomes are highly correlated to a provider’s expertise and commitment to assessment, triage, treatment, discharge, and adherence to given guidelines [14].

Several tools and strategies have been developed internationally to support obstetric triage. For example, the British Columbian Obstetric Triage Acuity Scale (OTAS) introduced a structured, five-level color-coded classification system that stratifies risk based on gestational age [15]. Similarly, traffic-light systems have been employed in sub-Saharan African labor wards to categorize patients according to clinical urgency [6,9]. These standardized tools have been shown to reduce unnecessary waiting times and improve diagnostic accuracy, contributing to more targeted and efficient care [6,9]. Nevertheless, their application is often inconsistent across settings, with few national-level guidelines available to ensure uniformity [16].

In Malawi, delays in receiving appropriate emergency care at health facilities are a leading contributor to perinatal mortality [17]. These delays often stem from challenges such as inadequate triage, shortages of equipment and drugs, poor blood supply, and overburdened staff of varying skill level [17]. Although many women seek care during emergencies, inefficient triage processes and poor recognition of obstetric danger signs significantly compromise outcomes [11,17,18].

Currently, Malawi has no national guidelines for obstetric triage, leaving admission decisions to labor ward staff. While WHO provides recommendations for intrapartum care, such as fetal heartbeat monitoring, vaginal examinations, companionship, and continuity of midwifery care, there are no explicit, context-adaptable guidelines for obstetric triage [19]. Existing literature emphasizes the clinical benefits of triage tools in improving clinical performance metrics, yet little attention has been given to the lived experiences of maternity care providers (MCPs), laboring women, and their companions [11,20–23].

While prior literature has established the importance of obstetric triage and standardized admission procedures in improving clinical efficiency and reducing emergency room delays, there is limited understanding of how these systems are experienced in the under-resourced maternity setting. Most literature has evaluated triage through measurable clinical outcomes, with less attention to how risk and danger are classified and interpreted by members of the delivery team. Furthermore, limited research has addressed how notions of risk, danger and purity deeply embedded in cultural and institutional logics influence the shaping of obstetric admission practices. This gap is especially relevant in contexts such as Malawi where national triage guidelines are absent and admission practices are instead locally developed routines. Therefore, this study seeks to understand how hospital-based admission procedures for childbirth in Malawi are experienced and understood by maternity care providers, laboring women, and their companions.

## Methods

### Ethics statement

Ethical approval was obtained from the Kamuzu University of Health Sciences Research and Ethics Committee (COMREC; P.04/20/3038) and the Swedish Ethical Review Authority (2020–01587). An amended version 3.1 was approved by the Kamuzu University College of Medicine Research and Ethics Committee (COMREC) on 11^th^ of November, 2020 to better gather information regarding the impact of health service changes and social/economic disruption due to COVID-19 on stakeholders experiences of maternity care at the district level. Written informed consent was obtained from all participants after they received study information and had the opportunity to ask questions. Information regarding the study’s purpose, procedures, potential risks, and confidentiality measures was provided. Participation was voluntary, participants could decline to answer any questions, and they could withdraw at any time without consequence. Anonymity and confidentiality were ensured by removing personal identifiers, assigning unique codes, restricting data access to authorized personnel, securely storing data in password-protected files, and reporting results in aggregate form.

### Study design

This qualitative research study utilized focus group discussions (FGDs), in-depth interviews, and observations to understand the standardization of obstetric admission and triaging in Malawian hospitals through the perspectives of pertinent maternity providers. This study was embedded within the larger Action Leveraging Evidence to Reduce Perinatal Mortality (ALERT) study which aimed to address regional discrepancies in perinatal outcomes in Benin, Uganda, Tanzania, and Malawi by targeting quality improvement interventions for mothers in the intrapartum period and their MCPs [24].

### Study setting

The Malawian healthcare system is divided into three provision agencies: 60% by the government through the Ministry of Health, ~ 40% by the Christian Health Association of Malawi (CHAM), with small contributions from private-for-profit centers. Healthcare is provided at the primary level- community hospitals, health centers, health posts, secondary level- district and faith-based hospitals, and tertiary level hospitals where all specialty care services are concentrated. All maternal-care related services are formally free at the point of care in governmental facilities and heavily subsidized at faith-based hospitals. However, only 20% of the population lives within 25 km of a healthcare center and physicians are only available in some secondary and tertiary facilities [25].

Data was collected from two rural hospitals in the central region; one being a faith-based facility under the CHAM designation and the other a public district hospital. The hospitals were chosen purposively based upon the specific inclusion criteria of a minimum caseload of 2500 births per year, cesarean section and blood transfusion services being available, location in a rural district, and inclusion of a mix of typical public and private-not-for-profit hospitals as representative of the Malawian healthcare landscape. The public district hospital provides free maternity care and serves a larger patient population, while the faith-based institution uses a fee-for-service model with fewer deliveries. The government hospital uses a two-shift staffing pattern, with approximately eight midwives per shift managing around 30 births each day. The faith-based hospital operated a three-shift system, with approximately four midwives per shift overseeing an average of 15 daily deliveries. Both facilities provide antenatal, intrapartum, postnatal, and neonatal services, as well as caesarean sections and blood transfusions. Companions were permitted throughout labor and birth in the faith-based hospital's private birthing rooms but were only beginning to be introduced in the public hospital as a result of improvements in ward privacy. Companions accompanied women to the facility and remained waiting outside even if they were not able to go in.

Maternity care is provided by nurses, midwives, nurse-midwives, midwifery technicians, and clinical officers. Training of the health workforce varies by profession, but includes certificate, diploma, and degree opportunities [26]. Staff-patient ratios pose challenges with only 0.4 doctors/10,000 individuals and 4.3 nurses/10,000 individuals [27]. Therefore, clinical officers perform 90% of cesarean sections [28].

Obstetric admissions and triaging at both hospitals are managed entirely by maternity providers, with expectant mothers presenting directly to the wards where they are received and assessed by MCPs. Hospital maternity wards serve a dual purpose; providing childbirth services to those who live nearby and referral care in case of complications [24]. Triage is conducted immediately upon arrival, based on clinical acuity, and continues to formal admission.

### Reflexivity

Reflexivity was embedded throughout this study through a collaborative research partnership that valued local expertise, interdisciplinary perspectives, and ongoing critical reflection. Conducted within the broader EU-funded ALERT consortium, the study addressed a locally relevant priority by examining hospital-based admission procedures for childbirth in Malawi to inform improvements in maternal care. A key strength of the study was the close collaboration with BK, whose deep understanding of the Malawian healthcare system, local context, and qualitative research methods was invaluable throughout the project. Together with WC and EC, BK established relationships with participating hospitals, collected the data, supervised the first author's fieldwork in Malawi, and made substantial contributions to the analysis and manuscript development. The research team included both clinical and non-clinical researchers, allowing different perspectives to inform the research while remaining mindful of how clinical authority could influence participant responses. The perceived neutrality of non-clinical researchers often encouraged more open conversations, and regular team discussions and critical reflection helped ensure that data collection and interpretation were shaped collaboratively rather than by any one perspective.

Reflexive dialogue was continuous throughout the study. During the first author's time in Malawi, preliminary coding and emerging interpretations were regularly discussed with BK and the local team before being refined through ongoing discussions with collaborators in Sweden. This iterative exchange was essential to challenging assumptions, incorporating local perspectives, and ensuring that interpretations remained grounded in the realities of the Malawian healthcare setting.

### Data collection

Data was collected between 25^th^, 2020, and 5^th^ January, 2021 using in-depth interviews, FDGs, and narrative observations to triangulate stakeholder’s perspectives regarding the standardization of obstetric admissions procedures. Participants included mothers, patient’s companions, and MCPs. Interview guides were used to conduct interviews and guide FGDs and were informed by all domains of the Afulani et al. (2019) Person-Centered Maternity Care Scale (PCMC) and the WHO guidelines on respectful care in an effort to assess clinical responsiveness using a validated questionnaire tool [24]. The hour-long interviews were based on open-ended questions and when participants for example, referred to supportive care or privacy, follow-up questions were asked to understand the experiences of the participants. The guides were piloted at a health center in Blantyre, Malawi, and adjusted to achieve data richness. MCPs and companions both were asked how they perceived the provision of maternity care at their facilities, while mothers were requested to recount the entirety of their obstetric experience from admission to discharge. Additionally, two sets of observations were performed at both hospitals by a skilled qualitative researcher, following women from their admission to discharge to document their care and workforce interactions across the length of their stay through detailed field notes, highlighting admission as a hotspot. The observer corroborated findings with working staff after each shift to ensure appropriate interpretation of events. Follow-up observations were conducted by the first author in April 2024 under the scope of the original COMREC approval. These observations were used to enhance contextual understanding of the everyday realities of maternity care in these settings. Immersion in the environment allowed the first author to observe how urgency was communicated through verbal and non-verbal interactions among maternity care providers, how the units were physically organized, including the distinct function of different spaces such as the latent waiting area, as well as some of the logistical challenges for laboring mothers such as the requirement to bring their own food and delivery supplies. Witnessing daily interactions between MCPs, other staff, the women, and their companions further illuminated workflows and communication practices that were not explicitly present within the transcripts alone as well as how overcrowding of the units impacted patient privacy and patient flow. Together, these experiences helped to ensure that emerging analyses remained grounded in the realities of the Malawian healthcare setting.

Malawi-based ALERT research assistants conducted the in-depth interviews with women and MCPs, facilitated the FGDs with companions and providers, performed the narrative observations, and completed the transcription and translation process to standardize data collection and prevent bias. The interviews were conducted in a mixture of English and Chichewa, a local language, in an effort to retain technical and cultural meaning and avoid interpretation gaps. Audio files and transcripts were tagged to protect interviewee anonymity and securely stored. Interviewers had a social science and reproductive health background and participated in face-to-face training in order to standardize interviewing techniques and facilitation measures. The research assistants regularly discussed emerging data and reflected upon the collection process.

### Participant sampling, recruitment, and characteristics

Purposive sampling based upon the principle of maximum variation was utilized for selection of women, MCPs, and companions within the study [29,30]. The title women is used to recognize that the participants comprised both mothers and primigravida. Data collection resulted in 22 in-depth interviews with women, 14 individual interviews with MCPs, two FGDs with companions, and two FGDs with MCPs (Tables 1–3). Information power determined sample saturation [29]. Inclusion criteria was defined for MCPs and required that both male and female maternity care providers covering both day and night shifts be included with varied years of work experience. Demographic characteristics were collected when available, however, incomplete information for some participants limited the extent to which maximum variation could be described across all characteristics.

**Table 1:** Participant mother demographic features.

Group: *Women*	Interviews	Age Range	Occupation	Parity	Education
Faith-Based Hospital	12	15–50 (NI: 1)	Business (1), Not Working (4), Farming (4), Student (1), Teacher (1), NI (1)	Primiparous (2), Multiparous (1–4) (5), Grand Multiparous (4), NI (1)	Primary (10), Tertiary (1), NI (1)
Public Hospital	10	17-46	Housewife (5), Business (3), Farming (2)	Primiparous (4), Multiparous (3), NI (3)	Primary (7), NI (3)

*NI: no information, IDI: Individual In-depth Interview, FGD: focus group discussion

**Table 2:** Participant maternity care provider demographic features.

Group: *Maternity Care Providers*	Interviews	Participants	Age Range	Occupation	Education
Faith-Based Hospital IDI	7	7	20–40	Nurse Midwife (2), Nurse (1), NMT (2), Clinical Officer (1), Nursing Officer (1)	Diploma (6), BSc (1)
Faith-Based Hospital FGD	1	4	24-39	Mixed maternity care provider cadres	NI
Public Hospital IDI	7	7	20–35 (NI: 1)	Intern (1), Nurse (3), Nursing Officer (1), Clinical Officer (1), NI (1)	Diploma (2), College (3), BSc (1), NI (1)
Public Hospital FGD	1	4	25–40	Nurse Midwife Tech (2), Nurse (1), Nursing Officer (1)	Diploma (3), BSc (1)

*NI: no information, IDI: Individual In-depth Interview, FGD: focus group discussion

**Table 3:** Participant companions demographic features.

Group: *Companions*	FGDs	Participants	Age Range	Occupation	Education
**Faith-Based Hospital**	1	6	35–60	Business (1), Farming (4), None (1)	Primary (4), Tertiary (1), None (1)
**Public Hospital**	1	7	35–60	Business (5), Farming (2)	Primary (5), None (2)

*NI: no information, IDI: Individual In-depth Interview, FGD: focus group discussion

### Reflexive thematic analysis

This study employed Reflexive Thematic Analysis (RTA), as outlined by Braun and Clarke, through inductive coding to explore stakeholder perspectives regarding obstetric admissions and triage procedures in Malawian hospitals [31]. Mary Douglas’ anthropological concepts of *purity*, *danger*, and *risk*—particularly as articulated in her seminal 1966 work *Purity and Danger*, provided a theoretical lens through which the analysis was informed [32]. Douglas’ framing of pregnancy as a liminal state, between being a woman and becoming a mother, highlights its symbolic and cultural ambiguity. In this context, obstetric procedures serve as formalized rites of passage designed to manage danger, eliminate disorder, and maintain symbolic and clinical “purity” [33]. This perspective aligns with how admissions and triaging are structured to identify risk and restore order in the maternity care process.

The analysis was conducted using NVivo 14, with all transcripts read and re-read to identify data segments relevant to the research focus. The coding and initial theme development were carried out by EB, under the supervision of HMA. Regular consultations with BK and WC, who were closely familiar with the study setting and participants, enriched the interpretation of cultural nuances and contextual complexities. This collaborative and reflexive process ensured a grounded and context-sensitive analysis. Sample size was guided by Malterud’s concept of information power [29]. This study had a focused aim to understand admission procedures within two specific rural maternity units in Malawi rather than generating population-level descriptions. Participants were purposively selected based on their direct involvement with the admissions process as they were authentically engaging with it. Douglas’ framework provided a theoretical lens to examine risk and danger within this space. Finally, the use of multiple qualitative methods and reflexive thematic analysis allowed the themes to be developed through repeated examination of the participant narratives and observed practice. Ultimately, themes were generated to capture and illuminate the diverse experiences of women, maternity care providers, and companions within the hospital admissions and triage process. Together, these elements provided sufficient information power to address the study aim.

### Patient and public involvement

Public involvement was not included in the design, conduct, reporting, or dissemination plans of the research. Transcripts were not returned to participants following translation by trained research assistants, and no subsequent member checking was conducted with participants during the research process. Therefore, while women and companions contributed their perspectives through interviews, their lack of involvement in shaping the research questions, analytic approach, or interpretation of findings may have limited the extent to which patient-centered understandings of obstetric admissions were captured.

## Results

THEME 1: **Hospital systems prioritize procedural consistency during admission. (**Table 4)

**Table 4:** Generated themes and subthemes.

Theme	Sub-Themes
*Overarching Theme: Institutional Processes Shape Risk, Authority, and Participation in Obstetric Admission*	• The overarching theme is informed by the following three themes and their subthemes.
Hospital systems prioritize procedural consistency during admission.	• The obstetric admissions form drives the admissions process.
Clinical risk is constructed through visible danger signs that organize obstetric decision-making.	• Reporting of danger signs is the basis for acuity-focused triaging procedures in obstetric admissions. • Shared understanding of danger signs reinforces the legitimacy of risk-based decision-making.
Selective communication reinforces professional authority while limiting patient and companion participation.	• MCPs economize what they share with patients, companions, and colleagues. • The sacrifice of personalized maternal care is recognized and normatively accepted by MCPs.

Obstetric admissions in Malawian hospitals were standardized through structured obstetric admission forms guiding triage and care. Despite slight variations across facilities, all forms included explicit assessment of danger signs including bleeding, fluid loss, severe pain, headache, and fever, pertinent obstetric history, obstetric physical examinations, and a targeted care plan. Although the forms themselves were not formal triage instruments, MCPs used the information collected to manage admissions, determine patient acuity, and direct clinical decisions.

The admission form’s pedagogic design allowed all levels of MCPs and students to assist with non-acute patient intake, particularly during chaotic periods of high patient loads. During observations, junior MCPs and students were often positioned at the admissions table where women first entered the clinical space. They completed the initial portions of the admissions form and gathered preliminary information, while the more senior MCP reviewed the assessment and made the final decision for disposition. Hospital staff recognized the utility of the admissions form in documenting the transition of the patient to be in the hands of the hospital, separated from their companions as they moved into a new phase.

MCPs adhered to admission protocols set by their unit matron, which included a prescribed sequence of questions and the use of four centimeter cervical dilation to distinguish active from latent labor. These standardized classifications transformed the uncertainty of a women’s arrival into clearly defined clinical categories that governed both admission and subsequent management. A female MCP highlighted this practice saying:

“*If they are in active phase of labor until delivery and they are four CM above you admit them, and you monitor fetal and maternal condition.”* (*MCP, Faith-Based Hospital)*

Observations across both facilities showed that admission extended beyond documentation to how patients moved through the maternity unit, with the standardized assessments, cervical dilation thresholds, and explicit danger signs screening questions determining when women were physically allowed to be in the labor ward.

Women in latent labor occupied an ambiguous clinical status, requiring more surveillance while awaiting sufficient progression to justify ward admission. MCPs used admission to a latent waiting area as a proactive mortality-reducing measure, housing high-risk mothers in early labor so they could be in close proximity to medical care instead of being sent home, where distance and transportation barriers could delay their return if labor progressed quickly or a complication arose. Although physically on hospital grounds, these women had not yet crossed the clinical threshold for labor ward admission, instead in an intermediary space with only intermittent observation as they awaited progression to active labor. Placement in the latent area reflected a clinical classification that balanced patient surveillance with delayed entry to the ward. At the public hospital, the latent waiting area was observed to be a tin-roofed cement structure located in the hospital courtyard, physically within the hospital but separate from the labor ward. Women and their companions remained in this intermediary space, storing their belongings, preparing food, and waiting for sufficient labor progression for ward admission. Patients and their companions often misunderstood the utility of utilizing the latent room for risk assessment, instead identifying the delay in ward admission as mistreatment, as they were only told to go and wait when the mother was not yet clinically ready to deliver. Companions felt particularly frustrated with this practice reflecting:

*“If they go to a latent room, if she feels pain say it is not yet time so they are suffering instead of helping us.” (Companion, Public Hospital)*

MCPs made strategic choices to separate the formality of the admissions process from the remainder of delivery, allowing maternity leadership to promote both patient safety and case load management during the most pressing periods. After the formal admissions process, the management of labor and delivery became highly pragmatic, shaped by patient circumstances, social factors, and available medical resources.

THEME 2: **Clinical risk is constructed through visible danger signs that organize obstetric decision-making. (Table 4)**

While the admissions process typically followed a standardized format, it was often adapted to respond more effectively to patient acuity, with providers frequently beginning medical intervention before completing the full admissions form.

*“Taking the history of the patient is a long process so to avoid losing the life of a patient we start intervening before a completion of history taking.” (MCP, Public Hospital)*

With high acuity mothers, a formal written admissions process was replaced by verbal questioning regarding danger signs and past birth complications to rapidly attend to her medical needs. Following stabilization or birth, the MCP returned to complete official documentation following the admissions form format.

The intentional placement of danger signs discussions early in the standardized admissions form intrinsically facilitated patient prioritization on the basis of acuity.

“*When a person comes like in a labour room she reaches on the station and we greet her and ask for danger signs. Danger signs are signs that the person is showing, e.g., losing blood and having a fast heartbeat.”* (*MCP, Faith-Based Hospital)*

Danger signs were among the first questions asked of the women, reflecting their role in early assessment. The absence of danger signs allowed women to progress through the routine admissions pathway, whereas the identification of danger signs immediately interrupted the standardized sequence, repositioning them into an emergency category that required more rapid intervention. Triaging through patient prioritization was used primarily during high patient volumes, particularly at the public hospital which was frequently crowded with more women than beds.

*“When the patients are many we do try to use triage because we only have few rooms and we may not take them all in.”* (*MCP, Public Hospital)*

Clinical classification extended beyond documentation to the physical organization of the labor ward, with women allocated to different spaces according to their assessed level of risk. The public hospital implemented specific additional triaging measures by assigning rooms for particular danger signs- pre-eclampsia, eclampsia, and hemorrhage.

Labor progression as assessed by physical examination was applied in conjunction with risk status in patient prioritization practices, with staff attention shifting from stable women to attend high-acuity and late phase deliveries.

*“It can happen that in the middle of assisting another patient, another woman can come with the labor pains, so you prioritize on which woman to assist, so that makes you to stop assisting the other and go assist the other.”*(*MCP, Faith-Based Hospital)*

MCPs routinely directed additional attention during admissions towards mothers with past poor outcomes to promote emotional care, “*They are below age or it is their sixth pregnancy but she has no child delivered alive so when you plan you need to try your best that she must deliver her baby.” (MCP, Faith-Based Hospital)*

Risk signs including severe bleeding, tachycardia, headache, and nausea/vomiting were widely recognized by maternity providers and formed the basis of rapid maternal and fetal assessment. MCPs prioritized clear communication about these signs with women and their companions as their primary risk-informed measure to prevent perinatal mortality. Upon admission, women were informed about danger signs and the steps needed to access care, and, understanding the urgency, they routinely complied with the questioning during the admissions process. Although companions were routinely informed about obstetric danger signs, they were rarely included in explanations regarding admission decisions, labor ward placement, or clinical management. Observations across both maternity units demonstrated that danger signs functioned as shared markers of obstetric risk among providers, women, and companions, visibly reorganizing the admissions process from routine assessment to emergency intervention when they were identified.

“*It is my duty as a guardian to remind the patient to go to the doctor and be examined. They will not send her back for when they go they examine them.” (Companion, Faith-Based Hospital)*

MCPs recognized danger signs as visible, measurable indicators for evaluating patient condition and prioritizing intervention within their contextual means as their examination was limited to basic physical techniques including digital vaginal exam and hand-held fetal scope.

THEME 3: **Selective communication reinforces professional authority while limiting patient and companion participation. (**Table 4)

Effective clinical communication was routinely sacrificed during admissions due to misunderstandings around preconceived care expectations and entrenched hierarchical norms.

A distinction existed between what was explicitly communicated with patients and companions and what was simply acted upon. MCPs neglected to introduce themselves, making it difficult for mothers to identify their care providers or formally report complaints. Consent for physical examination varied, with certain MCPs requesting permission and others proceeding without obtaining consent.

Following the mother’s initial greeting, the admissions process became largely interrogative, with patients unable to express any inquiries or concerns themselves. These one-sided interactions, while clinically efficient, encouraged blind trust in clinical authority. While MCPs recognized strong clinical communication as contributory to obstetric risk reduction, they routinely economized what they shared with patients, companions, and their colleagues.

*“Patients had little to say. Most of the time it was the nurse giving instructions or asking questions. The patient was mostly at the receiving end.” (Narrative Observations, Faith-Based Hospital)*

Companions were routinely excluded from discussions with providers during the admissions process yet were expected and encouraged to attend to the woman’s physical needs. While physically present within the maternity unit, their participation was largely confined to non-clinical roles, with staff legitimizing their exclusion by minimizing their involvement in patient care while recognizing their necessity for providing formal consent for cesarean delivery. This selective inclusion reinforced a clear boundary between those authorized to participate in clinical decision-making and those expected to support, not influence, the admissions process. Companions recognized the ultimate authority of providers, who often disregarded their suggestions regarding patient care, contributing to their silence during admissions.

*“They don’t consider guardians as important, they only value us when we come with the patient and when they want our consent but to be walking side by side in the whole process, no.” (Companion, Faith-Based Hospital)*

Infection-prevention rules in response to the COVID-19 pandemic affected how companions were able to physically access the labor units. At the faith based facility only one companion was allowed at a time and they were often sent out at night, told that they could infect the babies or sent to the kitchen outside, leaving the mothers alone.

*“Sometimes they also send us out at night. They say the patient will sleep alone. You should go and sleep at the kitchen, you may infect the babies with disease.” (Companion, Faith-Based Hospital)*

Companions also referenced the more direct route to the ward being closed due to COVID, making access the unit more difficult.

*“They should open up the way, but they said there is Corona, but sorry this corona they could sympathize with us companions.” (Companion, Faith-Based Hospital)*

Masking requirements and the implementation of tickets in order to enter the unit further intensified restrictions on companion movement and access.

*“For you to move from the kitchen to the ward to check on the patient, there are rules. They said there is a disease, we should always wear a mask. We wear a mask but for you to go through they give you a ticket. If you give them a ticket, you will get in.” (Companion, Faith-Based Hospital)*

MCPs reflected that they felt more able to emphasize risk-aversion through clinical communication when patients were well-mannered. They noted that companions appeared more comfortable, felt better informed, and were more likely to encourage the patient to comply with recommendations when they were included in discussions about the patient’s obstetric status and the need for admission.

Patients, companions, and staff alike viewed delays in admission as being dangerous, however MCPs felt unable to make meaningful changes beyond their protocols due to their facility’s critical contextual circumstances, resulting in often devastating outcomes.

*“But because they took time, that’s why the baby stopped breathing and died.” (Mother, Faith-Based Hospital)*

## Discussion

In Malawian maternity units, standardized admissions functioned as a system of classification by transforming the uncertainty of childbirth into ordered clinical categories. Drawing on Douglas’ concepts of purity and danger, these practices can be understood as institutional efforts to maintain social and clinical order by defining what constituted normal labor, obstetric risk, and emergency. While these classifications enabled providers to manage limited resources and prioritize urgent care, they also simultaneously created boundaries that shaped who belonged in particular spaces, whose knowledge was valued, and how women experienced care. This study revealed that obstetric admissions and triaging processes were standardized through a structured admissions form and clinical danger sign-based patient prioritization practices. While the admissions form guided initial intake, triage-based acuity assessments often superseded strict adherence to the form, improving patient flow and prioritizing care of high-risk mothers. Protocols for admission, cesarean indication with identified risk-factors, and acuity stratification bolstered MCPs’ clinical authority and provided a coping mechanism for them to address the intrinsic chaos of an under resourced obstetric ward through a reassured sense of psychological order and risk control. Standardization has been recognized in literature as a mechanism to promote safety and accuracy in healthcare, including the introduction of surgical timeout procedures in high-income settings, however their utility in managing high-volume obstetrics in low-resource contexts remains underexplored [34,35].

In contrast to structured obstetric triage models that emphasize systematic assessment, clear acuity categorization, timely reassessment, and patient-centered communication, the admission processes observed in these Malawian hospitals relied primarily on locally developed systems centered around danger sign recognition and clinical judgment. Tools such as the Obstetric Triage Acuity Scale (OTAS) and other standardized triage approaches seek to balance rapid identification of clinical urgency with consistent reassessment and communication across levels of care [15]. In these settings, however, formal triage systems were absent, and the admissions form functioned as the primary organizing framework. Although this approach enabled providers to rapidly identify high-risk patients within resource constraints, it provided fewer mechanisms for integrating patient preferences, emotional needs, or companion perspectives into early decision-making. Thus, the challenge was not the absence of standardization, but rather that existing classifications prioritized physiological risk while deprioritizing other dimensions of childbirth experience.

Universal recognition of danger signs by all obstetric stakeholders was further acknowledged as an imperative to address obstetric urgency and was therefore utilized as the foundation for triaging. Danger signs represented more than clinical indicators; they functioned as shared symbols through which providers, women, and companions categorized uncertainty and determined when routine childbirth required more intervention. Following guidelines and danger signs-informed triaging allowed MCPs to feel as though they were maximizing quality care despite challenging circumstances, additionally facilitating emotional separation from patient outcome responsibility. Knowledge regarding danger signs among expectant mothers and women of childbearing age has been evaluated at length in resource-limited settings including sub-Saharan Africa, with results of high parturient awareness [36–39]. Providers’ strong emphasis and reliance upon danger signs as the foundational mortality-reducing measure in turn enhanced awareness of symptoms by patients, companions, and MCPs alike, promoting a culture of risk and danger minimization. Many studies have specifically focused upon the awareness of companions to the importance of early recognition of danger signs in the prevention of mortality, particularly among men and male partners [40,41]. The factors contributing to MCPs’ reliance upon danger signs as the foundation for obstetric triaging practices is poorly documented, however their utility in the rapid identification of patient acuity has been affirmed [42,43]. Significant support given towards the importance of danger signs awareness and recognition suggests its validity as a cost-sensitive, life-saving measure to address preventable maternal and fetal death in low-resourced settings.

The results of this study highlight the dual impact of standardization: while it provided a necessary framework for maintaining order within unpredictable, resource-constrained maternity environments, it also reinforced boundaries that limited individualized care. Douglas argues that systems of classification create coherence but can also marginalize experiences that do not fit the established categories. In this setting, standardized admission procedures prioritized measurable clinical risk while leaving less space for emotional needs, patient preferences, and companion involvement. Standardization within healthcare has been recognized both as meaningful and obstructive, depending upon if they prioritize adaptability or sacrifice humanity, respectively [44]. Viewing routines from the patient perspective has been suggested as a measure to develop meaningful patient-centric practices [44]. Standardized care was understood to be more clinically beneficial for high acuity patients than individualized care, as clear mechanisms guaranteed that treatment was delivered systematically and efficiently [45]. Scholars, however, have acknowledged the conflict that exists between striving both for full standardization and full customization in healthcare, proposing individualized standardization as an alternative. Under this recommendation, care delivery is mutually informed by evidence and standardized protocols as well as by the perspectives and desires of the patients themselves [46–48]. Acknowledging the use of risk-aversion practices as justification for depersonalized care to maintain social order may support the adoption of individualized standardization as a meaningful approach to improve patient engagement and prioritize perceived quality of care in early delivery management. This study revealed how the ability for MCPs to offer patient centered obstetric care throughout the admissions process was largely contingent upon tacit agreements about what care should be provided, with women ultimately becoming the objectification of a work burden. Studies have validated effective clinical communication as critical to quality obstetric care, contributing to the reduction in preventable adverse obstetric outcomes and their controllable risk factors [49,50]. However, MCPs and parturients alike view the utility of communication differently as a result of social factors and pervasive power dynamics [51–56]. Provider and patient perceptions of shared decision-making have been evaluated in both high and low income contexts, with results suggesting the mutual preference for provider-led decisions, with some exceptions [57]. The legitimization of negating clinical communication in the promotion of risk-aversion suggests the prioritization of a clinical focus by MCPs in over-burdened obstetric settings. Hierarchical power dynamics prevented patients and companions from challenging their exclusion from decision-making, reinforcing a cycle where task-focused MCPs treated uninformed, passive patients. These communication practices reflected that MCPs maintained authority not only through their clinical expertise, but also through their ability to define which information was relevant to assessing risk and determining care. A 2020 Kenyan study similarly highlighted how a multitude of factors contribute to the neglect of psychological care and women’s autonomy in MCP communication [58]. Staff consciously involved women and companions primarily in legally significant situations, such as obtaining consent for cesarean delivery where poor consenting could be reprimanded. A scoping review on c-section counselling in sub-Saharan Africa found patient involvement and informed consent to be suboptimal, reflecting the legal emphasis on c-sections in these Malawian facilities [59]. A study in Benin suggested that parturient autonomy was sacrificed in the obstetric setting due to conditions of care provision, coercion, and women’s surrendering of autonomy [60].

Patients and companions support these findings, expressing that they often felt abandoned, perceiving that care was provided only when MCPs deemed it necessary or convenient. The combination of dismissive attitudes and the inability of the workforce to attend to individualized care as a result of intense workloads led to providers’ emotional detachment from their patients, focusing only on reducing risk through acute physical needs. In Southern Ethiopia, person-centered care was low, but positively associated with giving birth in a private facility and negatively impacted by parturient education and poor pregnancy outcomes [61]. The recent development of evidence-based guidance for psychosocial support during childbirth by the WHO has been implemented in low-resourced settings as a preliminary measure to address poor respectful maternity care (RMC) [62]. RMC alongside simple communication transparency could help reduce clinical miscommunication, which often frustrates MCPs, depersonalizes encounters, and increases the risk of adverse outcomes among this vulnerable population. Improving RMC in resource-constrained settings requires attention not only to the availability of clinical resources, but also to the classification systems that care is organized within. Therefore, while standardized procedures are essential for preventing avoidable mortality, systems must also recognize forms of knowledge that extend beyond explicitly clinical expertise. Incorporating women’s and companion’s perspectives into these processes may allow for labor units to preserve the safety of standardization while also reducing the unintended consequences of exclusion and depersonalization.

### Methodological Considerations- Strengths and Limitations

An understanding of obstetric admissions and triaging in Malawian hospitals was gained through triangulated data from interviews, FGDs, and narrative observations. Including both public and mission hospitals enhances result transferability, with purposive sampling of relevant obstetric staff and stakeholders strengthening the study’s credibility. Drawing on observations from both 2020/2021 and 2024 facilitated contextual re-immersion in the labor ward environment and strengthened the interpretation of participants’ accounts by situating them within the broader context of obstetric care. Social desirability bias may have influenced what the stakeholders shared with the data collectors as well as how the MCPs behaved during the admissions process as they were observed, however this was addressed by the research team returning several times over the course of the project to build rapport and trust with the participants. While maximum variation for sampling was attempted, availability of the patient population and logistically-based contextual factors limited its achievability [29,30].Companion involvement during the study period may have been influenced by both established practices and COVID-related infection-control measures; however, it is difficult to distinguish the effects of these factors within the data. Additionally, although the data collection tools were designed to focus on obstetric admission, they may not have fully captured all relevant aspects for this analysis, potentially overlooking valuable insights into admissions and triaging practices. Public involvement was not included in the design, conduct, reporting, or dissemination plans of the research. While women and companions contributed their perspectives through interviews, their lack of involvement in shaping the research questions, analytic approach, or interpretation of findings may have limited the extent to which patient-centered understandings of obstetric admissions were captured.

Finally, there was incomplete demographic information available for some of the participants, despite purposive selection to capture variation in clinical experiences and roles within the maternity care system. This may have limited our ability to fully characterize the sociodemographic diversity of our sample and constrained interpretation of how demographic factors influenced experiences of hospital-based admissions.

## Conclusions

In rural Malawian obstetric care, standardization was introduced as a necessary response to manage the uncertainty and risk of childbirth, promoting safety and order in unpredictable circumstances. Yet, this approach often depersonalized care, constrained women’s autonomy, and shaped hierarchical communication practices. These findings highlight the need for policies that balance clinical efficiency with more person-centered models of care. Potential approaches include modifying admission forms to incorporate communication and patient preference alongside risk assessment, establishing defined points for companion inclusion throughout the admission process, and developing in-service training that emphasizes respectful communication, cultural humility, and shared decision-making. Such adaptations may allow maternity units to maintain the benefits of standardized risk classification while preserving the human aspects of childbirth care.

## Supporting information

S1 Checklist Completed Inclusivity in Global Research Questionnaire describing considerations for equitable collaboration, local partnership, and representation in the conduct and reporting of research. (DOCX)

S2 Checklist Completed Standards for Reporting Qualitative Research (SRQR) checklist, with corresponding manuscript page numbers provided for each applicable reporting item. (DOCX)
