# Supplementary material for: Creating order in the uncertainty of childbirth: A qualitative study of obstetric admission and triage practices in rural Malawi
Source: PLOS Glob Public Health. 2026 Oct 26;6(10):e0006312. doi: 10.1371/journal.pgph.0006312 (PMC13704880; doi:10.1371/journal.pgph.0006312)
Supplement: Supplementary file 3 [file pgph.0006312.s003.docx]

**Peer Review**

**PLOS Global Public Health**

Manuscript: PGPH-D-26-01103

*“Addressing Risk and Danger During Maternity Triage in Central Malawian Hospitals”*

# Q1. Does this manuscript meet PLOS Global Public Health’s publication criteria?

**Recommendation: Major Revision**

The manuscript addresses an important and underexplored issue — how obstetric triage operates in resource-constrained Malawian hospitals and how standardisation interacts with respectful, person-centred care. The qualitative design is appropriate, the theoretical framing through Mary Douglas is original, and the topic aligns well with the journal’s scope. However, methodological, analytical, and reporting issues prevent publication in current form, and the conclusions are not always tightly anchored in the presented data.

# Q2. Has the statistical analysis been performed appropriately and rigorously?

Not directly applicable, this is qualitative research. Reflexive Thematic Analysis is named appropriately and the inductive coding approach is consistent with Braun and Clarke. However, three concerns about analytic rigor remain: (i) the Mary Douglas framework is invoked but the concept of “purity” never substantively appears in the results; (ii) the information power justification (Malterud) is asserted rather than demonstrated against the framework’s dimensions; (iii) PLOS journals expect adherence to a qualitative reporting checklist (COREQ or SRQR), none is provided, and several standard items are missing from the methods (interview duration, transcript return, member checking, interviewer credentials in the manuscript body).

# Q3. Have the authors made all data underlying the findings fully available?

**No, the data availability statement does not meet PLOS policy.** The current statement (“available from the corresponding author on reasonable request”) is insufficient. The authors should either justify a restriction explicitly (e.g., participant identifiability), name a non-author institutional contact, or at minimum deposit the interview guides, observation protocols, and final coding framework in a public repository (Figshare, Zenodo).

# Q4. Is the manuscript presented in an intelligible fashion and written in standard English?

The manuscript is generally readable but requires copy-editing. Specific issues:

- **Lines 71–74:** convoluted sentence about WHO guidance and “earlier efforts proved inadequate” (citation needed).
- **Line 318:** “interrogative questioning” is tautological.
- **Line 339:** “mothers initial greeting” missing apostrophe.
- **Line 360:** “MCPS” typo.
- **Tense** drifts between past and present in Results, standardise to past.
- **Reference 32** (a website on liminality) should be replaced with primary anthropological sources (Van Gennep, Turner).
- **References 7 and 9** appear to be Research Square preprints, replace with peer-reviewed versions if available.
- **Quote attribution** is inconsistent (some give role/gender/age/facility, others only role/facility).

# Q5. Review Comments to the Author

This manuscript presents qualitative findings on obstetric admissions and triage in two central Malawian hospitals (one CHAM, one public), drawing on 36 in-depth interviews, 4 FGDs, and ward observations. The authors argue that admissions are standardised through structured forms and danger-sign-led triage; that this enables care under high patient volumes but at the cost of personalised, communicative care; and that companions and women themselves are largely excluded from decision-making. The topic is timely and important, and the bi-institutional design is a real strength. The following revisions would substantially strengthen the manuscript.

## Major concerns

1. ***Unaddressed pandemic context.*** Primary data collection occurred 25 November 2020 – 5 January 2021, the height of the COVID-19 pandemic in southern Africa. The manuscript contains no acknowledgement of how the pandemic may have shaped companion access, provider workload, infection-driven depersonalisation, or staffing. A 2024 follow-up observation is mentioned but its findings are not reported in any depth. The authors should either explicitly position findings as pandemic-era and treat this as a contextual factor and limitation, or integrate the 2024 follow-up data substantively to show which patterns persist. As written, readers cannot disentangle pandemic dynamics from structural features of Malawian obstetric care.
2. ***Weak integration of the theoretical framework.*** The Mary Douglas lens is prominent in the title, abstract, and methods, but the results read as a descriptive account of triage practice. “Purity” never substantively appears in the results, and the “liminality” framing is asserted but not analytically traced. The authors should either deepen the theoretical interpretation (e.g., showing how protocol adherence functions as purification, how the latent room operates as a liminal space, how companion exclusion enacts symbolic boundary maintenance) or reduce the theoretical claim and reframe the study as one informed by rather than structured by Douglas’s work.
3. ***Sub-theme substantiation is uneven.*** Theme 1’s second sub-theme on the “individual unit matron” is named in Table 4 and briefly mentioned at line 246, but is not developed. Either expand with quotes/observations or remove. Narrative observation data are cited only minimally, despite being one of three data sources.
4. ***Sample composition and demographic gaps.*** Tables 1–3 contain extensive “NI” entries (e.g., three of ten public-hospital women have no recorded age, parity, or occupation). For a study claiming maximum-variation purposive sampling, these gaps undermine the variation claim. Provide the missing data or explain why it could not be obtained and how this affects the diversity represented.
5. ***Discussion is disproportionately literature-driven.*** The Discussion (lines 371–452) frequently introduces findings from other studies before foregrounding the authors’ own. The Discussion is also notably longer than the Results, suggesting under-presentation of data. Trim literature comparison and expand data extracts in Results.
6. ***Vague conclusions.*** The conclusion calls for “rebalancing clinical priorities with communication and emotional support” but offers no concrete recommendations directed at policy actors, training institutions, or facility management. Given the study’s stated aim to inform context-adaptable triage, the authors should propose specific, actionable adaptations — for example, modifications to the admission form, defined points for companion inclusion, or specific in-service training content.

## Minor concerns

- ***Patient and Public Involvement*** (line 217): the absence of PPI is a notable limitation in a study about respectful maternity care; acknowledge it in the limitations section, not only as a stand-alone statement.
- ***Ethics:*** confirm whether the 2024 follow-up observation was covered by the original COMREC approval or a separate amendment.
- ***Faith-based vs. public comparison:*** the study includes both facility types but rarely contrasts them. Are there differences in admission practices, companion inclusion, or communication norms between CHAM and government facilities? If yes, develop this; if no, state so explicitly.
- ***Reflexivity:*** the section (lines 149–155) defers detail to “supplementary material” that appears to contain only ethics approvals. Given that the first author is based in Sweden analysing Malawian data, a substantive reflexivity statement on positionality, power dynamics in coding, and cross-cultural interpretation should appear in the manuscript itself.
- ***Table 4*** could be strengthened by adding a third column with one representative quote per sub-theme.
- ***Line 162:*** clarify whether the entire PCMC tool informed the interview guides, or specific domains.
- ***Lines 131–134:*** clarify whether both facilities are rural; the text is inconsistent on this point.

## Concerns about ethics, dual publication, or competing interests

No evidence of dual publication or competing interests. Ethical approvals from COMREC (P.04/20/3038) and the Swedish Ethical Review Authority (2020-01587) are documented. Please confirm IRB coverage of the 2024 follow-up observation.
