# Supplementary material for: Creating order in the uncertainty of childbirth: A qualitative study of obstetric admission and triage practices in rural Malawi
Source: PLOS Glob Public Health. 2026 Oct 26;6(10):e0006312. doi: 10.1371/journal.pgph.0006312 (PMC13704880; doi:10.1371/journal.pgph.0006312)
Supplement: Supplementary file 4 [file pgph.0006312.s004.docx]

**Review of Article**

**Summary**

Overall, the manuscript explores an important and under researched topic with great impact especially in the Malawian context. It can offer meaningful contribution to existing literature. However, a clear articulation of the objectives and sharp conclusion is lacking. The mention of what the study adds to existing literature needs to be strongly built in. The use of theoretical lens is not brought clearly. Results lack clear hierarchy of themes. Interpretation of results is missing. Conclusions need to be tightly linked to findings.

**Major comments**

- Clarity on the research question - The study manuscript should clearly articulate the research question and the result should be linked with it.
- The gap in the literature that this study is addressing needs proper articulation once the research question is framed.
- Reflexive thematic analysis however mentioned is not visible in the result section.
- Results are described in detail but sharp interpretations with tightened conclusions will add more meaning.

**Minor comments**

**Abstract**

The title is broad can be more specific,abstract can be tighter to reflect on findings.

**Introduction**

The aims and objectives of the study lack clarity and differ in the abstract and the main background. While the study chooses to study an important area with establishing importance of triaging in reducing burden of perinatal mortality, the review of literature does not follow the gap the study is going to address.

For example – at the end of background section, the study reads “this study seeks to interrogate how danger is identified, interpreted, and managed during the maternity triage process in under-resourced Malawian hospitals” while the study design mentions that the study seeks to “understand the standardization of obstetric admission and triaging in Malawian hospitals through the perspectives of pertinent maternity providers” whereas the abstract section mentions that the study aims to understand the processes.

The researcher needs to maintain the consistency of objectives with which the study was planned throughout.

References have been cited in the background without clear engagements of the content. Objectives and the result of 2024 follow up is not clear in the study and was it planned in between the study or from beginning to be clarified.

**Methods**

- Details on inclusion or exclusion criteria can be added. Theoretical lens mentioned earlier needs to be used consistently.
- Consent procedures can de written in detail.

**Results**

- Details on the danger signs whether being part of the standardized admission format or not can bring clarity on the use of danger as inherent part of format or by the staff.
- The study has subtly captured that the formats that are used are not triaging forms but rather regular admission forms which forces MCPs to stop filling it when they identify the presence of danger signs, when the case is actually risk assessment using the format.
- The theme and the resulting result section does not follow. While the theme table mentions about “MCPs economize what they share with patients, companions, and colleagues.” Also result reads that patient misunderstood the latent room use. The text reads that the companions were well informed, this creates misleading results.
- The table of theme does not utilises Mary Douglas’ *Purity and Danger*, and it is not clear how theme generation followed this concept except for danger signs.
- The study establishes a clear practice; structured admission process with a superseded acuity based assessment. It is important to compare it to what should be an ideal process or what is prescribed by standardised risk assessment tools that are mentioned in the background section (WHO and other tools).

**Discussion and conclusions**

- There is discussion on findings from other studies, for example education level of patients, power dynamics, alternative medicine, etc. have not studied or emerged in present study which questions the need of this articles references.
- The strength quoted as longitudinal perspective has not brought in the result section.
- Conclusions need to be very tight and linked to the objectives.
