# Supplementary material for: Creating order in the uncertainty of childbirth: A qualitative study of obstetric admission and triage practices in rural Malawi
Source: PLOS Glob Public Health. 2026 Oct 26;6(10):e0006312. doi: 10.1371/journal.pgph.0006312 (PMC13704880; doi:10.1371/journal.pgph.0006312)
Supplement: Supplementary file 5 [file pgph.0006312.s006.docx]

***PLOS Global Public Health Revisions***

*Creating Order in the Uncertainty of Childbirth: A Qualitative Study of Obstetric Admission and Triage Practices in Rural Malawi*

| ***PLOS Comment*** | ***Manuscript Revision*** |
| --- | --- |
| 1. Please include a complete copy of PLOS’ questionnaire on inclusivity in global research in your revised manuscript. Our policy for research in this area aims to improve transparency in the reporting of research performed outside of researchers’ own country or community. The policy applies to researchers who have travelled to a different country to conduct research, research with Indigenous populations or their lands, and research on cultural artefacts. The questionnaire can also be requested at the journal’s discretion for any other submissions, even if these conditions are not met. Please find more information on the policy and a link to download a blank copy of the questionnaire here: <https://journals.plos.org/globalpublichealth/s/best-practices-in-research-reporting>. Please upload a completed version of your questionnaire as Supporting Information when you resubmit your manuscript. | Please find the PLOS questionnaire uploaded within “supporting information”. |
| 2. Please ensure that your Ethics Statement is available in its entirety at the beginning of your Methods section, under a subheading 'Ethics Statement'. | Please find the ethics statement at the beginning of the Methods section of the revised manuscript. |
| 3. Please amend your detailed Financial Disclosure statement. This is published with the article. It must therefore be completed in full sentences and contain the exact wording you wish to be published.  a. State the initials, alongside each funding source, of each author to receive each grant. b. State what role the funders took in the study. If the funders had no role in your study, please state: “The funders had no role in study design, data collection and analysis, decision to publish, or preparation of the manuscript.” c. If any authors received a salary from any of your funders, please state which authors and which funders. | Please find the financial disclosure amended with the funder role and salary for each author as requested.  “This study is part of the ALERT project, funded by the European Commission's Horizon 2020. The research was funded by ALERT (No 847824, PI Claudia Hanson), under a call for implementation research for maternal and child health, and the Swedish Research Council (project number 2020–04949). The funders had no role in study design, data collection and analysis, decision to publish, or preparation of the manuscript.”  **HMA, CH, BK:** salary from both ALERT and the Swedish Research Council  **EC**: salary from ALERT |
| 4. Your manuscript is missing the following sections: Introduction. Please ensure these are present, and in the correct order, and that any references to subheadings in your main text are correct. An outline of the required sections can be consulted in our submission guidelines here: [LINK]  <https://journals.plos.org/water/s/submission-guidelines#loc-parts-of-a-submission>  <https://journals.plos.org/globalpublichealth/s/submission-guidelines#loc-parts-of-a-submission> | Please find the sections previously titled “background” changed to “introduction” in both the abstract and main text. |
| 5. We have noticed that you have uploaded Supporting Information files, but you have not included a list of legends. Please add a full list of legends for your Supporting Information files after the references list | Please find a list of legends at the end of the revised main text. |
| 6. In the online submission form, you indicated that "The transcripts used and/or analyzed during the current study are available from the corresponding author on reasonable request.".  All PLOS journals now require all data underlying the findings described in their manuscript to be freely available to other researchers, either  1. In a public repository,  2. Within the manuscript itself, or  3. Uploaded as supplementary information.  This policy applies to all data except where public deposition would breach compliance with the protocol approved by your research ethics board. If your data cannot be made publicly available for ethical or legal reasons (e.g., public availability would compromise patient privacy), please explain your reasons by return email and your exemption request will be escalated to the editor for approval. Your exemption request will be handled independently and will not hold up the peer review process, but will need to be resolved should your manuscript be accepted for publication. One of the Editorial team will then be in touch if there are any issues. | Please find this response included within the online submission form.  **Data are available upon reasonable request**. Data can be accessed upon reasonable request. The manuscript and its supporting information files contain all pertinent data. The qualitative primary data, including transcripts, capture the perspectives of maternity care providers from two healthcare facilities in Malawi, where the provider population is quite small. Publicly releasing the full data set could compromise the privacy assurances given to participants. Our ethical approvals were contingent on maintaining participant anonymity. Consequently, the full transcripts cannot be shared. However, specific text excerpts will be reviewed for identifying details and made available to researchers or reviewers who agree to a data- sharing agreement and adhere to strict confidentiality protocols. For data requests, contact: claudia.hanson@ ki.se. |
| **Figure Revision:**  While revising your submission, we strongly recommend that you use PLOS’s NAAS tool (<https://ngplosjournals.pagemajik.ai/artanalysis>) to test your figure files. NAAS can convert your figure files to the TIFF file type and meet basic requirements (such as print size, resolution), or provide you with a report on issues that do not meet our requirements and that NAAS cannot fix.  After uploading your figures to PLOS’s NAAS tool - <https://ngplosjournals.pagemajik.ai/artanalysis>, NAAS will process the files provided and display the results in the "Uploaded Files" section of the page as the processing is complete. If the uploaded figures meet our requirements (or NAAS is able to fix the files to meet our requirements), the figure will be marked as "fixed" above. If NAAS is unable to fix the files, a red "failed" label will appear above. When NAAS has confirmed that the figure files meet our requirements, please download the file via the download option, and include these NAAS processed figure files when submitting your revised manuscript. | Thank you for your comments. The manuscript only have tables, no figures and therefore we respectfully will just retain them. |
| **Reviewer #1:**  Q1. The manuscript addresses an important and underexplored issue how obstetric triage operates in resource-constrained Malawian hospitals and how standardization interacts with respectful, person-centered care. The qualitative design is appropriate, the theoretical framing through Mary Douglas is original, and the topic aligns well with the journal’s scope. However, methodological, analytical, and reporting issues prevent publication in current form, and the conclusions are not always tightly anchored in the presented data. | Please find these general comments addressed in subsequent edits below. |
| Q2. Has the statistical analysis been performed appropriately and  rigorously?  Not directly applicable, this is qualitative research. Reflexive Thematic Analysis is named  appropriately and the inductive coding approach is consistent with Braun and Clarke. However,  three concerns about analytic rigor remain: (i) the Mary Douglas framework is invoked but the  concept of “purity” never substantively appears in the results; (ii) the information power  justification (Malterud) is asserted rather than demonstrated against the framework’s  dimensions; (iii) PLOS journals expect adherence to a qualitative reporting checklist (COREQ or  SRQR), none is provided, and several standard items are missing from the methods (interview  duration, transcript return, member checking, interviewer credentials in the manuscript body). | Thank you for these important comments!   1. Concept of purity in the results:   “These standardized classifications transformed the uncertainty of a women’s arrival into clearly defined clinical categories that governed both admission and subsequent management.”   2. Demonstrate information power   Sample size was guided by  “Sample size was guided by Malterud’s concept of information power (28). This study had a focused aim to understand admission procedures within two specific rural maternity units in Malawi rather than generating population-level descriptions. Additionally, participants were purposively selected based on their direct involvement with the admissions process as they were authentically engaging with it. Douglas’ framework provided a theoretical lens to examine risk, danger within this space. Finally, the use of multiple qualitative methods and reflexive thematic analysis allowed the themes to be developed through repeated examination of the participant narratives and observed practice. Ultimately, themes were generated to capture and illuminate the diverse experiences of women, maternity care providers, and companions within the hospital admissions and triage process. Together, these elements provided sufficient information power to address the study aim.”   3. SRQR checklist with points addressed   Please find the SRQR checklist attached within the supplementary material. |
| Q3. Have the authors made all data underlying the findings fully  available??  No, the data availability statement does not meet PLOS policy. The current statement  (“available from the corresponding author on reasonable request”) is insufficient. The authors  should either justify a restriction explicitly (e.g., participant identifiability), name a non-author  institutional contact, or at minimum deposit the interview guides, observation protocols, and final  coding framework in a public repository (Figshare, Zenodo). | **Data are available upon reasonable request**. Data can be accessed upon reasonable request. The manuscript and its supporting information files contain all pertinent data. The qualitative primary data, including transcripts, capture the perspectives of maternity care providers from two healthcare facilities in Malawi, where the provider population is quite small. Publicly releasing the full data set could compromise the privacy assurances given to participants. Our ethical approvals were contingent on maintaining participant anonymity. Consequently, the full transcripts cannot be shared. However, specific text excerpts will be reviewed for identifying details and made available to researchers or reviewers who agree to a data- sharing agreement and adhere to strict confidentiality protocols. For data requests, contact: claudia.hanson@ ki.se. |
| Q4. Is the manuscript presented in an intelligible fashion and written  in standard English?  The manuscript is generally readable but requires copy-editing. Specific issues:  • Lines 71–74: convoluted sentence about WHO guidance and “earlier efforts proved  inadequate” (citation needed).  • Line 318: “interrogative questioning” is tautological.  • Line 339: “mothers initial greeting” missing apostrophe.  • Line 360: “MCPS” typo.  • Tense drifts between past and present in Results, standardise to past.  • Reference 32 (a website on liminality) should be replaced with primary anthropological  sources (Van Gennep, Turner).  • References 7 and 9 appear to be Research Square preprints, replace with peer-  reviewed versions if available.  • Quote attribution is inconsistent (some give role/gender/age/facility, others only  role/facility). | Lines 71-74: Please find these lines revised “In contrast, many low-and middle income countries (LMICs) lack comparable standardized approached, as global guidance has not yet provided context specific recommendations for obstetric triage and previous implementation efforts have demonstrated limited success. (<https://pmc.ncbi.nlm.nih.gov/articles/PMC12399223/>)”  Line 318: “Interrogative questioning” has been replaced with “questioning”  Line 339: Please find an apostrophe added to “mothers”.  Line 360: Please find “MCPS” edited to “MCPs”.  Tense: The results text has been verified to be in past tense.  Reference 32: Please find the reference replaced with the primary text by Van Gennep in the revised manuscript.  References 7 and 9: Please find the references updated to reference open access research articles.  Quote attribution: please find the quote attributions amended to list only role and facility as that information is available for all entries. |
| 1. Unaddressed pandemic context. Primary data collection occurred 25 November 2020 – 5 January 2021, the height of the COVID-19 pandemic in southern Africa. The manuscript contains no acknowledgement of how the pandemic may have shaped companion access, provider workload, infection-driven depersonalization, or staffing. A 2024 follow-up observation is mentioned but its findings are not reported in any depth. The authors should either explicitly position findings as pandemic-era and treat this as a contextual factor and limitation, or integrate the 2024 follow-up data substantively to show which patterns persist. As written, readers cannot disentangle pandemic dynamics from structural features of Malawian obstetric care. | Thank you for bringing up this important point regarding the influence of the COVID pandemic on obstetric experience. Please find formal clarification within the ethics statement:  “An amended version 3.1 was approved by the Kamuzu University College of Medicine Research and Ethics Committee (COMREC) on 11^th^ of November, 2020 to better gather information regarding the impact of the health service changes and social/economic disruption due to COVID-19 on stakeholders experiences of maternity care at the district level.”  Please also find additional findings and quotations to support included within the results section:  Infection-prevention rules in response to the COVID-19 pandemic affected how companions were able to physically access the labor units. At the faith based facility only one companion was allowed at a time and they were often sent out at night, told that they could infect the babies or sent to the kitchen outside, leaving the mothers alone.    *“Sometimes they also send us out at night. They say the patient will sleep alone. You should go and sleep at the kitchen, you may infect the babies with disease.” (Companion, Faith-Based Hospital)*  Companions also referenced the more direct route to the ward being closed due to COVID, making access the unit more difficult.    *“They should open up the way, but they said there is Corona, but sorry this corona they could sympathize with us companions.” (Companion, Faith-Based Hospital)*    Masking requirements and the implementation of tickets in order to enter the unit further intensified restrictions on companion movement and access.    *“For you to move from the kitchen to the ward to check on the patient, there are rules. They said there is a disease, we should always wear a mask. We wear a mask but for you to go through they give you a ticket. If you give them a ticket, you will get in.” (Companion, Faith-Based Hospital)* |
| 2. Weak integration of the theoretical framework. The Mary Douglas lens is prominent in the title, abstract, and methods, but the results read as a descriptive account of triage practice. “Purity” never substantively appears in the results, and the “liminality” framing is asserted but not analytically traced. The authors should either deepen the theoretical interpretation (e.g., showing how protocol adherence functions as purification, how the latent room operates as a liminal space, how companion exclusion enacts symbolic boundary maintenance) or reduce the theoretical claim and reframe the study as one informed by rather than structured by Douglas’s work. | We really appreciate you highlighting the importance of significantly integrating Douglas’s framework. Please find examples of sentences that have been added to achieve this.   - “Observations across both facilities showed that admission extended beyond documentation to how patients moved through the maternity unit, with the standardized assessments, cervical dilation thresholds, and danger signs screening questions determining when women were physically allowed to be in the labor ward.” - “Although physically within the hospital, these women had not yet crossed the clinical threshold for labor ward admission, instead in an intermediary space with intermittent observation as they awaited progression to active labor. Placement in the latent area reflected a clinical classification that balanced patient surveillance with delayed entry to the ward.” - “This selective inclusion reinforced a clear boundary between those authorized to participate in clinical decision-making and those expected to support, not influence, the admissions process.” - “Women in latent labor occupied an ambiguous clinical status, requiring more surveillance while awaiting sufficient progression to justify ward admission.” - “The absence of danger signs allowed women to progress through the routine admissions pathway, whereas the identification of danger signs immediately interrupted the standardized sequence, repositioning them into an emergency category that required more rapid intervention.” - “Clinical classification extended beyond documentation to the physical organization of the labor ward, with women allocated to different spaces according to their assessed level of risk.” - “Observations across both maternity units demonstrated that danger signs functioned as shared markers of obstetric risk among providers, women, and companions, visibly reorganizing the admissions process from routine assessment to emergency intervention when they were identified.” |
| 3. Sub-theme substantiation is uneven. Theme 1’s second sub-theme on the “individual unit matron” is named in Table 4 and briefly mentioned at line 246, but is not developed. Either expand with quotes/observations or remove. Narrative observation data are cited only minimally, despite being one of three data sources. | Thank you very much for bringing up this important comment. Please find Theme 1’s second theme removed and several examples of narrative observations added to the results as shown below:     - “Observations across both facilities showed that admission extended beyond documentation to how patients moved through the maternity unit, with the standardized assessments, cervical dilation thresholds, and danger signs screening questions determining when women were physically allowed to be in the labor ward.” - “Observations across both maternity units demonstrated that danger signs functioned as shared markers of obstetric risk among providers, women, and companions, visibly reorganizing the admissions process from routine assessment to emergency intervention when they were identified.” - “During observations, junior MCPs and students were often positioned at the admissions table where women first entered the clinical space. They completed the initial portions of the admissions form and gathered preliminary information, while the more senior MCP reviewed the assessment and made the final decision for disposition.” - “At the public hospital, the latent waiting area was observed to be a tin-roofed cement structure located in the hospital courtyard, physically within the hospital but separate from the labor ward. Women and their companions remained in this intermediary space, storing their belongings, preparing food, and waiting for sufficient labor progression for ward admission.” |
| 4. Sample composition and demographic gaps. Tables 1–3 contain extensive “NI” entries (e.g., three of ten public-hospital women have no recorded age, parity, or occupation). For a study claiming maximum-variation purposive sampling, these gaps undermine the variation claim. Provide the missing data or explain why it could not be obtained and how this affects the diversity represented. | Thank you for noting this discrepancy. Please find additional clarification in both the methods and limitations:  Methods:  “Demographic characteristics were collected when available, however, incomplete information for some participants limited the extent to which maximum variation could be described across all characteristics.”  Limitations:  “Finally, there was incomplete demographic information available for some of the participants, despite purposive selection to capture variation in clinical experiences and roles within the maternity care system. This may have limited our ability to fully characterize the sociodemographic diversity of our sample and constrained interpretation of how demographic factors influenced experiences of hospital-based admissions.” |
| 5. Discussion is disproportionately literature-driven. The Discussion (lines 371–452) frequently introduces findings from other studies before foregrounding the authors’ own. The Discussion is also notably longer than the Results, suggesting under-presentation of data. Trim literature comparison and expand data extracts in Results. | Thank you for recognizing the need for further expansion of the results. More data and commentary has been added to the results section and the discussion has been modified to more closely align with Douglas’ theory.  “In Malawian maternity units, standardized admissions functioned as a system of classification by transforming the uncertainty of childbirth into ordered clinical categories. Drawing on Douglas’ concepts of purity and danger, these practices can be understood as institutional efforts to maintain social and clinical order by defining what constituted normal labor, obstetric risk, and emergency. While these classifications enabled providers to manage limited resources and prioritize urgent care, they also simultaneously created boundaries that shaped who belonged in particular spaces, whose knowledge was valued, and how women experienced care.”  “Danger signs represented more than clinical indicators; they functioned as shared symbols through which providers, women, and companions categorized uncertainty and determined when routine childbirth required more intervention.”  “The results of this study highlight the dual impact of standardization: while it provided a necessary framework for maintaining order within unpredictable, resource-constrained maternity environments, it also reinforced boundaries that limited individualized care. Douglas argues that systems of classification create coherence but can also marginalize experiences that do not fit the established categories. In this setting, standardized admission procedures prioritized measurable clinical risk while leaving less space for emotional needs, patient preferences, and companion involvement.”  “Improving RMC in resource-constrained settings requires attention not only to the availability of clinical resources, but also to the classification systems that care is organized within. Therefore, while standardized procedures are essential to prevent avoidable mortality, systems must also recognize forms knowledge that are not explicitly clinical. Incorporating women’s and companion’s perspectives into these processes may allow for labor units to preserve the safety of standardization while also reducing the unintended consequences of exclusion and depersonalization.” |
| 6. Vague conclusions. The conclusion calls for “rebalancing clinical priorities with communication and emotional support” but offers no concrete recommendations directed at policy actors, training institutions, or facility management. Given the study’s stated aim to inform context-adaptable triage, the authors should propose specific, actionable adaptations — for example, modifications to the admission form, defined points for companion inclusion, or specific in-service training content. | Thank you so much for your specific suggestions! Please find some additional commentary added to the conclusion.  “Potential approaches include modifying admission forms to incorporate communication and patient preference alongside risk assessment, establishing defined points for companion inclusion throughout the admission process, and developing in-service training that emphasizes respectful communication, cultural humility, and shared decision-making. Such adaptations may allow maternity units to maintain the benefits of standardized risk classification while preserving the human aspects of childbirth care.” |
| ***Minor concerns***  • Patient and Public Involvement (line 217): the absence of PPI is a notable limitation in  a study about respectful maternity care; acknowledge it in the limitations section, not  only as a stand-alone statement. | Thank you for the comment! Please find the following included within the public and public involvement section:  “While women and companions contributed their perspectives through interviews, their lack of involvement in shaping the research questions, analytic approach, or interpretation of findings may have limited the extent to which patient-centered understandings of obstetric admissions were captured.” |
| • Ethics: confirm whether the 2024 follow-up observation was covered by the original  COMREC approval or a separate amendment. | Thank you for requesting clarification. Please find additional sentences within the methods:  “Follow-up observations were conducted by the first author in April 2024 under the scope of the original COMREC approval. These observations were used to enhance contextual understanding of the everyday realities of maternity care in these settings. Immersion in the environment allowed the first author to observe how urgency was communicated through verbal and non-verbal interactions among maternity care providers, how the units were physically organized, including the distinct function of different spaces such as the latent waiting area, as well as some of the logistical challenges for laboring mothers such as the requirement to bring their own food and delivery supplies. Witnessing daily interactions between MCPs, other staff, the women, and their companions further illuminated workflows and communication practices that were not explicitly present within the transcripts alone as well as how overcrowding of the units impacted patient privacy and patient flow. Together, these experiences helped to ensure that emerging analyses remained grounded in the realities of the Malawian healthcare setting.” |
| • Faith-based vs. public comparison: the study includes both facility types but rarely  contrasts them. Are there differences in admission practices, companion inclusion, or  communication norms between CHAM and government facilities? If yes, develop this; if  no, state so explicitly. | Thank you for providing the opportunity for further explanation of the study setting. Please find the expanded description below:  “The public district hospital provides free maternity care and serves a larger patient population, while the faith-based institution uses a fee-for-service model with fewer deliveries. The government hospital uses a two-shift staffing pattern, with approximately eight midwives per shift managing around 30 births each day. The faith-based hospital operates a three-shift system, with approximately four midwives per shift overseeing an average of 15 daily deliveries. Both facilities provide antenatal, intrapartum, postnatal, and neonatal services, as well as caesarean sections and blood transfusions. Companions are permitted throughout labor and birth in the faith-based hospital's private birthing rooms but not in the government hospital's shared labor ward.” |
| • Reflexivity: the section (lines 149–155) defers detail to “supplementary material” that  appears to contain only ethics approvals. Given that the first author is based in Sweden  analyzing Malawian data, a substantive reflexivity statement on positionality, power dynamics in coding, and cross-cultural interpretation should appear in the manuscript  itself. | Thank you for bringing up this important point for further clarification. Please find the amended language as included in the updated manuscript below:  “Reflexivity was embedded throughout this study through a collaborative research partnership that valued local expertise, interdisciplinary perspectives, and ongoing critical reflection. Conducted within the broader EU-funded ALERT consortium, the study addressed a locally relevant priority by examining hospital-based admission procedures for childbirth in Malawi to inform improvements in maternal care. A key strength of the study was the close collaboration with BK, whose deep understanding of the Malawian healthcare system, local context, and qualitative research methods was invaluable throughout the project. Together with WC and EC, BK established relationships with participating hospitals, collected the data, supervised the first author's fieldwork in Malawi, and made substantial contributions to the analysis and manuscript development. The research team included also included both clinical and non-clinical researchers, allowing different perspectives to inform the research while remaining mindful of how clinical authority could influence participant responses. The perceived neutrality of non-clinical researchers often encouraged more open conversations, and regular team discussions and critical reflection helped ensure that data collection and interpretation were shaped collaboratively rather than by any one perspective.  Reflexive dialogue was continuous throughout the study. During the first author's time in Malawi, preliminary coding and emerging interpretations were regularly discussed with BK and the local team before being refined through ongoing discussions with collaborators in Sweden. This iterative exchange was essential to challenging assumptions, incorporating local perspectives, and ensuring that interpretations remained grounded in the realities of the Malawian healthcare setting.” |
| Table 4 could be strengthened by adding a third column with one representative quote  per sub-theme. | We thank the reviewer for this helpful suggestion. We agree that participant quotations are important for demonstrating the grounding of themes in the data. However, we decided not to add a representative quote for each sub-theme in the results table because this would considerably increase the size of the table and reduce its function as a concise summary of the findings. To maintain readability and adhere to space considerations, we instead present illustrative quotations in the main Results text, where sufficient context and interpretation can be provided. We believe this approach allows readers to assess the connection between the data and the analytical themes while preserving the clarity of the summary table. |
| Line 162: clarify whether the entire PCMC tool informed the interview guides, or specific  domains. | Thank you for the comment. Please find the amended language in the updated manuscript: “Interview guides were used to conduct interviews and guide FGDs and were informed by all domains of the Afulani et al. (2019) Person-Centered Maternity Care Scale (PCMC) and the WHO guidelines on respectful care in an effort to assess clinical responsiveness using a validated questionnaire tool (23). The interviews were based on open-ended questions and when participants for example, referred to supportive care or privacy, follow-up questions were asked to understand the experiences of the participants.” |
| Lines 131–134: clarify whether both facilities are rural; the text is inconsistent on this  point. | Thank you for the request for clarification. Please find that rurality has been clarified in the following text:  “Data was collected from two **rural** hospitals in the central region; one being a faith-based facility under the CHAM designation and the other a public district hospital. The hospitals were chosen purposively based upon the specific inclusion criteria of a minimum caseload of 2500 births per year, cesarean section and blood transfusion services being available, **location in a rural district**, and inclusion of a mix of typical public and private-not-for-profit hospitals as representative of the Malawian healthcare landscape.” |
| **Reviewer #2:** |  |
| Clarity on the research question - The study manuscript should clearly articulate the  research question and the result should be linked with it. | Thank you for this point of clarification. Please find the research question revised to: “How are hospital-based admission procedures for childbirth in Malawi experienced and understood by maternity care providers, laboring women, and their companions?” within the updated manuscript. |
| The gap in the literature that this study is addressing needs proper articulation once the  research question is framed. | Thank you for bringing this important point to our attention. Please find additional information regarding the gap in literature articulated within the introduction.  “While prior literature has established the importance of obstetric triage and standardized admission procedures in improving clinical efficiency and reducing emergency room delays, there is limited understanding of how these systems are socially constructed and experienced in the under-resourced maternity setting. Most literature has evaluated triage through measurable clinical outcomes, with less attention to how risk and danger are classified and interpreted by members of the delivery team. Furthermore, limited research has addressed how notions of risk, danger and purity deeply embedded in cultural and institutional logics influence the shaping of obstetric admission practices. This gap is especially relevant in contexts such as Malawi where national triage guidelines are absent and admission practices are instead locally developed routines. Therefore, this study seeks to understand how hospital-based admission procedures for childbirth in Malawi are experienced and understood by maternity care providers, laboring women, and their companions.” |
| Reflexive thematic analysis, however mentioned, is not visible in the result section. | Please find that the themes have been amended to be more interpretive and reflective of the reflexive thematic analysis methodology.  Theme 1: Hospital systems prioritize procedural consistency during admission.  Theme 2: Clinical risk is constructed through visible danger signs that organize obstetric decision-making.  Theme 3: Selective communication reinforces professional authority while limiting patient and companion participation. |
| Results are described in detail but sharp interpretations with tightened conclusions will add  more meaning. | Thank you for this important comment! The themes have been reformulated to illustrate the interpretive process with conclusions clarified.  Theme 1: Hospital systems prioritize procedural consistency during admission.  Theme 2: Clinical risk is constructed through visible danger signs that organize obstetric decision-making.  Theme 3: Selective communication reinforces professional authority while limiting patient and companion participation.  Conclusions:  “Potential approaches include modifying admission forms to incorporate communication and patient preference alongside risk assessment, establishing defined points for companion inclusion throughout the admission process, and developing in-service training that emphasizes respectful communication, cultural humility, and shared decision-making. Such adaptations may allow maternity units to maintain the benefits of standardized risk classification while preserving the human aspects of childbirth care.” |
| *Abstract*  The title is broad can be more specific, abstract can be tighter to reflect on findings. | Thank you for your comment. Please find both the title and abstract revised to better reflect the results and conclusions.  Creating Order in the Uncertainty of Childbirth: A Qualitative Study of Obstetric Admission and Triage Practices in Rural Malawi  **Introduction**  Maternal and neonatal mortality continues to be a concern in sub-Saharan Africa. Although standardized obstetric triage improves clinical outcomes, little is know about how locally developed admission practices function in low-resource settings where national obstetric triage guidelines are absent. This study explored how hospital-based obstetric admission procedures in rural Malawi were experienced by maternity care providers, laboring women, and companions.    **Methods**  We used qualitative methods including in-depth interviews with mothers and maternity care providers, focus group discussions with companions and maternity care providers, and observations of two labor wards. Study participants were chosen purposively to represent diverse birth outcomes and professionals. Data analysis followed reflexive thematic analysis, concentrating on obstetric admissions. Mary Douglas’ concepts of purity, danger, and risk informed interpretation during theme development.  **Results**  Obstetric admissions were organized through standardized admission forms that structured assessment and enabled providers to classify women according to visible danger signs and labor progression. Although the forms were not formal triage tools, they functioned as the primary mechanism for prioritizing care, organizing patient movement through maternity spaces, and managing high patient volumes. Shared recognition of danger signs among providers, women, and companions reinforced the legitimacy of risk-based decision making. At the same time, communication was selectively restricted, limiting patient and companion participation while reinforcing professional authority and hierarchical relationships.  **Conclusion**  Standardized admission procedures created order by classifying obstetric risk and prioritizing clinical efficiency, but they also reinforced hierarchical relationships that limited communication and patient participation. Efforts to strengthen obstetric admissions should preserve the benefits of standardized risk assessment while incorporating more person-centered communication and companion engagement. |
| *Introduction*  The aims and objectives of the study lack clarity and differ in the abstract and the main background.  While the study chooses to study an important area with establishing importance of triaging in  reducing burden of perinatal mortality, the review of literature does not follow the gap the study is going to address. For example – at the end of background section, the study reads “this study seeks to interrogate  how danger is identified, interpreted, and managed during the maternity triage process in under-  resourced Malawian hospitals” while the study design mentions that the study seeks to “understand  the standardization of obstetric admission and triaging in Malawian hospitals through the  perspectives of pertinent maternity providers” whereas the abstract section mentions that the study  aims to understand the processes. | Please find the abstract and introduction aims more closely aligned with the perspective of understanding rather than interrogating.  “Therefore, this study seeks to understand how hospital-based admission procedures for childbirth in Malawi are experienced and understood by maternity care providers, laboring women, and their companions.” |
| The researcher needs to maintain the consistency of objectives with which the study was planned  throughout. | Please find the manuscript aims more closely aligned with the perspective of understanding admission processes as described below:  “Therefore, this study seeks to understand how hospital-based admission procedures for childbirth in Malawi are experienced and understood by maternity care providers, laboring women, and their companions.” |
| Objectives and the result of 2024 follow up is not clear in the study and was it planned in between the study or from the beginning to be clarified. | Thank you for requesting this point of clarification. Please find additional sentences added within the methods:  “Follow-up observations were conducted by the first author in April 2024 under the scope of the original COMREC approval. These observations were used to enhance contextual understanding and evaluate changes in departmental practices over time.” |
| *Methods*   Details on inclusion or exclusion criteria can be added. Theoretical lens mentioned earlier  needs to be used consistently. | Thank you for providing the opportunity for further clarification of methodology as written below:  Inclusion criteria was defined for MCPs and required that both male and female maternity care providers covering both day and night shifts be included with varied years of work experience. |
| Consent procedures can be written in detail. | Thank you for bringing this point to our attention. Please find detailed consent information within the “ethics statement” section of methods.  “Written informed consent was obtained from all participants after they received study information and had the opportunity to ask questions. Information regarding the study’s purpose, procedures, potential risks, and confidentiality measures was provided. Participation was voluntary, participants could decline to answer any questions, and they could withdraw at any time without consequence.” |
| *Results*   Details on the danger signs whether being part of the standardized admission format or not can bring clarity on the use of danger as inherent part of format or by the staff. | Thank you for this comment. Please find this clarified in two spots.  “Despite slight variations across facilities, all forms included **explicit assessment of danger signs including bleeding, fluid loss, severe pain, headache, and fever**, pertinent obstetric history, obstetric physical examinations, and a targeted care plan. Maternity care providers used these forms to manage admissions and direct clinical decisions.”  “Observations across both facilities showed that admission extended beyond documentation to how patients moved through the maternity unit, with the standardized assessments, cervical dilation thresholds, and **explicit danger signs screening questions** determining when women were physically allowed to be in the labor ward.” |
| The study has subtly captured that the formats that are used are not triaging forms but  rather regular admission forms which forces MCPs to stop filling it when they identify the  presence of danger signs, when the case is actually risk assessment using the format. | Thank you for this insightful observation. Please find this explicitly addressed within the results as amended:  “Although the forms themselves were not formal triage instruments, MCPs used the information collected to manage admissions, determine patient acuity, and direct clinical decisions.” |
| The theme and the resulting result section does not follow. While the theme table mentions  about “MCPs economize what they share with patients, companions, and colleagues.” Also  result reads that patient misunderstood the latent room use. The text reads that the  companions were well informed, this creates misleading results. | Thank you for providing the opportunity for clarification! Please find the statement hopefully clarified.  “Although companions were routinely informed about obstetric danger signs, they were rarely included in explanations regarding admission decisions, labor ward placement, or clinical management.” |
| The table of theme does not utilize Mary Douglas’ Purity and Danger, and it is not clear how  theme generation followed this concept except for danger signs. | Please find that the themes have been amended to more closely align with Douglas and Reflexive Thematic Analysis.  Hospital systems prioritize procedural consistency during admission.  Clinical risk is constructed through visible danger signs that organize obstetric decision-making.  Selective communication reinforces professional authority while limiting patient and companion participation. |
| The study establishes a clear practice; structured admission process with a superseded  acuity based assessment. It is important to compare it to what should be an ideal process or  what is prescribed by standardized risk assessment tools that are mentioned in the  background section (WHO and other tools). | Thank you for your comment. Please find a paragraph added to the discussion which goes into more detail regarding this topic.  “In contrast to structured obstetric triage models that emphasize systematic assessment, clear acuity categorization, timely reassessment, and patient-centered communication, the admission processes observed in these Malawian hospitals relied primarily on locally developed systems centered around danger sign recognition and clinical judgment. Tools such as the Obstetric Triage Acuity Scale (OTAS) and other standardized triage approaches seek to balance rapid identification of clinical urgency with consistent reassessment and communication across levels of care (14). In these settings, however, formal triage systems were absent, and the admissions form functioned as the primary organizing framework. Although this approach enabled providers to rapidly identify high-risk patients within resource constraints, it provided fewer mechanisms for integrating patient preferences, emotional needs, or companion perspectives into early decision-making. Thus, the challenge was not the absence of standardization, but rather that existing classifications prioritized physiological risk while deprioritizing other dimensions of childbirth experience.” |
| Discussion and conclusions   There is discussion on findings from other studies, for example education level of patients,  power dynamics, alternative medicine, etc. have not studied or emerged in present study  which questions the need of this articles references. | Thank you for your comment. Please find the sources discussing education level and alternative medicine removed from the manuscript. |
| The strength quoted as longitudinal perspective has not brought in the result section. | Thank you for recognizing the need for more integration of longitudinal perspective. We hope the addition of more narrative observations within the results to be satisfactory. |
| Conclusions need to be very tight and linked to the objectives. | Please find that the conclusions have been tightened both within the discussion and final conclusion.  “Improving RMC in resource-constrained settings requires attention not only to the availability of clinical resources, but also to the classification systems that care is organized within. Therefore, while standardized procedures are essential to prevent avoidable mortality, systems must also recognize forms knowledge that are not explicitly clinical. Incorporating women’s and companion’s perspectives into these processes may allow for labor units to preserve the safety of standardization while also reducing the unintended consequences of exclusion and depersonalization.”  “In rural Malawian obstetric care, standardization was introduced as a necessary response to manage the uncertainty and risk of childbirth, promoting safety and order in unpredictable circumstances. Yet, this approach often depersonalized care, constrained women’s autonomy, and shaped hierarchical communication practices. These findings highlight the need for policies that balance clinical efficiency with more person-centered models of care. Potential approaches include modifying admission forms to incorporate communication and patient preference alongside risk assessment, establishing defined points for companion inclusion throughout the admission process, and developing in-service training that emphasizes respectful communication, cultural humility, and shared decision-making. Such adaptations may allow maternity units to maintain the benefits of standardized risk classification while preserving the human aspects of childbirth care.” |
