# Supplementary material for: Creating order in the uncertainty of childbirth: A qualitative study of obstetric admission and triage practices in rural Malawi
Source: PLOS Glob Public Health. 2026 Oct 26;6(10):e0006312. doi: 10.1371/journal.pgph.0006312 (PMC13704880; doi:10.1371/journal.pgph.0006312)
Supplement: Supplementary file 6 [file pgph.0006312.s007.docx]

***PLOS Global Public Health Revisions***

*Creating Order in the Uncertainty of Childbirth: A Qualitative Study of Obstetric Admission and Triage Practices in Rural Malawi*

| **PLOS Comment** | **Manuscript Revision** |
| --- | --- |
| Data were collected between November 2020 and January 2021, and the revision now demonstrates that infection-control rules directly shaped companion access, which is central to the paper's third theme. The limitations section, however, contains no mention of COVID-19. Please state plainly there that companion exclusion in particular is likely to reflect both structural features of Malawian obstetric care and pandemic-specific restrictions, and that the two cannot be fully separated with these data. | Thank you for your important comment. Please find additional clarification within the limitations section. “Companion involvement during the study period may have been influenced by both established practices and COVID-related infection-control measures; however, it is difficult to distinguish the effects of these factors within the data.” |
| Relatedly, the limitations section continues to claim a longitudinal perspective showing the evolution of practices, but the observation extracts are not dated or attributed to 2020/21 versus April 2024, so no temporal comparison is visible to the reader. Please either substantiate the claim by stating which patterns persisted in 2024 and labelling extracts by period, or soften it to what it actually is: a contextual re-immersion that supported interpretation. | Please find that the language has been softened to recognize the 2024 observations as a reimmersion. “Drawing on observations from both 2020/2021 and 2024 facilitated contextual re-immersion in the labor ward environment and strengthened the interpretation of participants’ accounts by situating them within the broader context of obstetric care.” |
| Changes to Tables 1 and 2. Previously missing demographic data have been filled in. Public-hospital women's age range moves from "20-35 (NI: 3)" to "17-46" with the NI notation dropped, occupations are recoded from "Not Working (2), Business (3), Farming (2), NI (3)" to "Housewife (5), Business (3), Farming (2)", and the faith-based FGD row gains an age range and cadre description where it previously showed NI throughout. Parity still shows NI (3). Meanwhile the methods and limitations now state that demographic information was incomplete. Please explain in the methods how these data were subsequently obtained, and reconcile the tables with the new limitation statement. If the recoding of "Not Working" to "Housewife" reflects a reinterpretation rather than newly recovered data, please say so; the two terms are not equivalent and the change alters how the sample's variation reads. | Thank you for this comment. While some of the information was able to be further deciphered, certain elements still do not have information. Therefore, it is still truthful within the methods and limitations section to state that demographic information was incomplete. |
| The methods still describe Douglas as providing the lens for theme formation and retain the rites-of-passage paragraph, while the results operationalize classification and boundary maintenance but not purity or pollution. Either state explicitly in the methods that the framework informed rather than structured the analysis, or add one or two sentences making the purity dimension analytically visible, for example in how the latent room separates the not-yet-clinically-legitimate from the admitted. | Thank you for your comment. Please find that the verbiage has been modified to “informed”.  “Mary Douglas’ anthropological concepts of *purity*, *danger*, and *risk*—particularly as articulated in her seminal 1966 work *Purity and Danger*, provided a theoretical lens through which the analysis was informed.” |
| Note also that Table 4's overarching theme, "The Imposition of Standardized Obstetric Admissions and Triage", was not updated when the three sub-themes were reworded and now reads in a different register from them. | Please find the overarching theme to be updated: ***Institutional Processes Shape Risk, Authority, and Participation in Obstetric Admission.*** |
| Reporting items in the methods body. Interview duration, whether transcripts were returned to participants, and whether any member checking took place are still absent from the manuscript text. | Thank you for the comment. Please find the data collection and patient and public involvement sections updated to reflect the appropriate changes.  “Transcripts were not returned to participants following translation by trained research assistants, and no subsequent member checking was conducted with participants during the research process.” |
| Internal contradiction on companion access. The new study setting paragraph states that companions were permitted in the faith-based hospital's private birthing rooms but not in the government hospital's shared labor ward. A sentence retained later still reads that companions "were being introduced at the public hospital following improvements in labor ward privacy". Please reconcile these; the second sentence appears superseded. Since a companion focus group was conducted at the public hospital, it would also help to clarify that companions accompanied women to that facility even where they could not enter the ward. | Thank you for addressing this contradiction. Please find that a single statement has been refined to reflect the concept instead.  “Companions were permitted throughout labor and birth in the faith-based hospital's private birthing rooms but were only beginning to be introduced in the public hospital as a result of improvements in ward privacy. Companions accompanied women to the facility and remained waiting outside even if they were not able to go in.” |
| The new sentence acknowledging the absence of PPI as a constraint remains inside the standalone PPI section. Please relocate or duplicate it into the limitations section. | Please find that the patient and public involvement section has been duplicated within the limitations section. |
| Abstract: "little is know about how..." - this also appears in the abstract entered into the submission system, which is what is typeset, so it needs correcting in both places. | Please find “know” edited to “known” in both the manuscript and submission system. |
| Reflexivity: "The research team included also included both clinical and non-clinical researchers." | Please find “also included” deleted to maintain appropriate grammar of the sentence. |
| Analysis section: "a theoretical lens to examine risk, danger within this space" - missing conjunction. | Please find the addition of “and” within the sentence to fill the missing conjunction. |
| Discussion: "systems must also recognize forms knowledge that are not explicitly clinical" - missing word. | Please find this sentence as a replacement:  “Therefore, while standardized procedures are essential for preventing avoidable mortality, systems must also recognize forms of knowledge that extend beyond explicitly clinical expertise.” |
| Introduction: "many low-and middle income countries" - hyphenation. | Please find an additional hyphen added between middle and income. |
| Results: one residual present tense, "the admissions process becomes largely interrogative". | Please find “becomes” replaced with “became” to maintain past tense. |
| Introduction, line 96: the citation (16) should be (17). The sentence on inadequate triage, equipment shortages, blood supply and overburdened staff draws on Mgawadere et al., now reference 17; reference 16 is the Rashidi Fakari narrative review on triage scales. | Please find the citation updated to 17 to reflect the Mgawadere source. |
| Limitations, line 612: the citation (28-29) should be (29-30) for Malterud and Palinkas, consistent with the methods. | Please find the citation updated to 29-30 in order to align with the methods and Malterud/Palinkas. |
