# Supplementary material for: Creating order in the uncertainty of childbirth: A qualitative study of obstetric admission and triage practices in rural Malawi
Source: PLOS Glob Public Health. 2026 Oct 26;6(10):e0006312. doi: 10.1371/journal.pgph.0006312 (PMC13704880; doi:10.1371/journal.pgph.0006312)
Supplement: Peer review history

# Peer review: Decision Letter 0

**DOI:** 10.1371/journal.pgph.0006312.r001

**Authors:** Jennifer Tucker

22 Jun 2026

PGPH-D-26-01103

Addressing Risk and Danger During Maternity Triage in Central Malawian Hospitals

PLOS Global Public Health

Dear Dr. Bundy,

Thank you for submitting your manuscript to PLOS Global Public Health. After careful consideration, we feel that it has merit but does not fully meet PLOS Global Public Health’s publication criteria as it currently stands. Therefore, we invite you to submit a revised version of the manuscript that addresses the points raised during the review process.

Your manuscript has been reviewed by two reviewers, who have recommended revision of your manuscript. Please address all comments in your revised submission.

Please submit your revised manuscript by Aug 18 2026 11:59PM. If you will need more time than this to complete your revisions, please reply to this message or contact the journal office at globalpubhealth@plos.org. When you're ready to submit your revision, log on to https://www.editorialmanager.com/pgph/ and select the 'Submissions Needing Revision' folder to locate your manuscript file.

Please include the following items when submitting your revised manuscript:

- A letter that responds to each point raised by the editor and reviewer(s). You should upload this letter as a separate file labeled 'Response to Reviewers'.
- A marked-up copy of your manuscript that highlights changes made to the original version. You should upload this as a separate file labeled 'Revised Manuscript with Track Changes'.
- An unmarked version of your revised paper without tracked changes. You should upload this as a separate file labeled 'Manuscript'.

Guidelines for resubmitting your figure files are available below the reviewer comments at the end of this letter.

As the corresponding author, your ORCID iD is verified in the submission system and will appear in the published article. PLOS supports the use of ORCID, and we encourage all coauthors to register for an ORCID iD and use it as well. Please encourage your coauthors to verify their ORCID iD within the submission system before final acceptance, as unverified ORCID iDs will not appear in the published article. *Only*  the individual author can complete the verification step; PLOS staff *cannot*  verify ORCID iDs on behalf of authors.

We look forward to receiving your revised manuscript.

Kind regards,

Jennifer Tucker, PhD

Staff Editor

PLOS Global Public Health

Journal Requirements:

1. Please include a complete copy of PLOS’ questionnaire on inclusivity in global research in your revised manuscript. Our policy for research in this area aims to improve transparency in the reporting of research performed outside of researchers’ own country or community. The policy applies to researchers who have travelled to a different country to conduct research, research with Indigenous populations or their lands, and research on cultural artefacts. The questionnaire can also be requested at the journal’s discretion for any other submissions, even if these conditions are not met. Please find more information on the policy and a link to download a blank copy of the questionnaire here: https://journals.plos.org/globalpublichealth/s/best-practices-in-research-reporting. Please upload a completed version of your questionnaire as Supporting Information when you resubmit your manuscript.

2. Please ensure that your Ethics Statement is available in its entirety at the beginning of your Methods section, under a subheading 'Ethics Statement'.

3. Please amend your detailed Financial Disclosure statement. This is published with the article. It must therefore be completed in full sentences and contain the exact wording you wish to be published.

a. State the initials, alongside each funding source, of each author to receive each grant.

b. State what role the funders took in the study. If the funders had no role in your study, please state: “The funders had no role in study design, data collection and analysis, decision to publish, or preparation of the manuscript.”

c. If any authors received a salary from any of your funders, please state which authors and which funders.

4. Your manuscript is missing the following sections: Introduction. Please ensure these are present, and in the correct order, and that any references to subheadings in your main text are correct. An outline of the required sections can be consulted in our submission guidelines here: [LINK]

https://journals.plos.org/water/s/submission-guidelines#loc-parts-of-a-submission

https://journals.plos.org/globalpublichealth/s/submission-guidelines#loc-parts-of-a-submission

5. We have noticed that you have uploaded Supporting Information files, but you have not included a list of legends. Please add a full list of legends for your Supporting Information files after the references lis

6. In the online submission form, you indicated that "The transcripts used and/or analyzed during the current study are available from the corresponding author on reasonable request.".

All PLOS journals now require all data underlying the findings described in their manuscript to be freely available to other researchers, either

1. In a public repository,

2. Within the manuscript itself, or

3. Uploaded as supplementary information.

This policy applies to all data except where public deposition would breach compliance with the protocol approved by your research ethics board. If your data cannot be made publicly available for ethical or legal reasons (e.g., public availability would compromise patient privacy), please explain your reasons by return email and your exemption request will be escalated to the editor for approval. Your exemption request will be handled independently and will not hold up the peer review process, but will need to be resolved should your manuscript be accepted for publication. One of the Editorial team will then be in touch if there are any issues.

If the reviewer comments include a recommendation to cite specific previously published works, please review and evaluate these publications to determine whether they are relevant and should be cited. There is no requirement to cite these works unless the editor has indicated otherwise.

Additional Editor Comments (if provided):

[Note: HTML markup is below. Please do not edit.]

Reviewers' comments:

Reviewer's Responses to Questions

**Comments to the Author**

1. Does this manuscript meet PLOS Global Public Health’s publication criteria?

Reviewer #1: Yes

Reviewer #2: Yes

**********

2. Has the statistical analysis been performed appropriately and rigorously?

Reviewer #1: N/A

Reviewer #2: I don't know

**********

3. Have the authors made all data underlying the findings in their manuscript fully available (please refer to the Data Availability Statement at the start of the manuscript PDF file)?

The PLOS Data policy

Reviewer #1: No

Reviewer #2: Yes

**********

4. Is the manuscript presented in an intelligible fashion and written in standard English?

Reviewer #1: Yes

Reviewer #2: Yes

**********

Reviewer #1: This manuscript presents qualitative findings on obstetric admissions and triage practices in two central Malawian hospitals (one CHAM faith-based facility, one public district hospital), drawing on 36 in-depth interviews, 4 focus group discussions, and ward observations. The authors argue that admissions are standardised through structured forms and danger-sign-led triage, that this standardisation enables care under high patient volumes but at the cost of personalised, communicative care, and that companions and women themselves are largely excluded from decision-making. Mary Douglas's Purity and Danger is invoked as a theoretical lens.

Reviewer #2: The manuscript is an original research. It can offer meaningful contribution. There are some major revisions needed, clear articulation of objectives, and sharp conclusion. There is need to built in what the study will add to the existing literature. Results need to add clear hierarchy of themes. If these are addressed it can be accepted for publication.

**********

what does this mean?). If published, this will include your full peer review and any attached files.

**Do you want your identity to be public for this peer review?**  If you choose “no”, your identity will remain anonymous but your review may still be made public.

For information about this choice, including consent withdrawal, please see our Privacy Policy

Reviewer #1: No

Reviewer #2: No

**********

[NOTE: If reviewer comments were submitted as an attachment file, they will be attached to this email and accessible via the submission site. Please log into your account, locate the manuscript record, and check for the action link "View Attachments". If this link does not appear, there are no attachment files.]

Attachment Submitted filename: Peer_Review_PGPH-D-26-01103.docx

Attachment Submitted filename: Review of Article PLOS.docx

# Author comment: Author response to Decision Letter 1

**DOI:** 10.1371/journal.pgph.0006312.r002

14 Aug 2026

Attachment Submitted filename: Response to Reviewers.docx

# Peer review: Decision Letter 1

**DOI:** 10.1371/journal.pgph.0006312.r003

**Authors:** Helen Howard

14 Sep 2026

PGPH-D-26-01103R1

Creating Order in the Uncertainty of Childbirth: A Qualitative Study of Obstetric Admission and Triage Practices in Rural Malawi

PLOS Global Public Health

Dear Dr. Bundy,

Thank you for submitting your manuscript to PLOS Global Public Health. After careful consideration, we feel that it has merit but does not fully meet PLOS Global Public Health’s publication criteria as it currently stands. Therefore, we invite you to submit a revised version of the manuscript that addresses the points raised during the review process.

Please submit your revised manuscript by Nov 10 2026 11:59PM. If you will need more time than this to complete your revisions, please reply to this message or contact the journal office at globalpubhealth@plos.org. When you're ready to submit your revision, log on to https://www.editorialmanager.com/pgph/ and select the 'Submissions Needing Revision' folder to locate your manuscript file.

Please include the following items when submitting your revised manuscript:

- A letter that responds to each point raised by the editor and reviewer(s). You should upload this letter as a separate file labeled 'Response to Reviewers'.
- A marked-up copy of your manuscript that highlights changes made to the original version. You should upload this as a separate file labeled 'Revised Manuscript with Track Changes'.
- An unmarked version of your revised paper without tracked changes. You should upload this as a separate file labeled 'Manuscript'.

Guidelines for resubmitting your figure files are available below the reviewer comments at the end of this letter.

As the corresponding author, your ORCID iD is verified in the submission system and will appear in the published article. PLOS supports the use of ORCID, and we encourage all coauthors to register for an ORCID iD and use it as well. Please encourage your coauthors to verify their ORCID iD within the submission system before final acceptance, as unverified ORCID iDs will not appear in the published article. *Only*  the individual author can complete the verification step; PLOS staff *cannot*  verify ORCID iDs on behalf of authors.

We look forward to receiving your revised manuscript.

Kind regards,

Helen Howard

Staff Editor

PLOS Global Public Health

**Journal Requirements:**

If the reviewer comments include a recommendation to cite specific previously published works, please review and evaluate these publications to determine whether they are relevant and should be cited. There is no requirement to cite these works unless the editor has indicated otherwise.

**Additional Editor Comments (if provided):**

[Note: HTML markup is below. Please do not edit.]

Reviewers' comments:

Reviewer's Responses to Questions

**Comments to the Author**

Reviewer #1: All comments have been addressed

**********

publication criteria?

Reviewer #1: Partly

**********

3. Has the statistical analysis been performed appropriately and rigorously?

Reviewer #1: N/A

**********

4. Have the authors made all data underlying the findings in their manuscript fully available (please refer to the Data Availability Statement at the start of the manuscript PDF file)?

The PLOS Data policy

Reviewer #1: Yes

**********

5. Is the manuscript presented in an intelligible fashion and written in standard English?

Reviewer #1: Yes

**********

**Reviewer #1:**  This is a substantive and largely faithful revision. I checked each claim in the point-by-point response against the revised manuscript text, and the great majority of the promised changes are genuinely present. The reframed title, research question and abstract are a clear improvement, and the aims are now consistent across abstract, introduction and methods. The results are better grounded in narrative observation data, particularly the admissions-table hierarchy and the description of the latent waiting area. The reference problems raised previously have been fixed properly: both preprints are replaced with the peer-reviewed versions, the liminality website is replaced with van Gennep, all 62 references are present and cited, and the references orphaned by the removal of the education-level and alternative-medicine material were cleanly deleted rather than left dangling.

Two changes deserve particular credit. First, the authors have accepted the observation that the instruments used are admission forms rather than triage tools, and have stated this explicitly in the results and abstract instead of defending the original framing. This makes the paper's central argument sharper. Second, the new discussion paragraph contrasting the local system with OTAS and other structured triage models, and concluding that the issue is not the absence of standardization but what the existing classification prioritizes, is the strongest new material in the manuscript.

The following points remain.

1. Pandemic context and the 2024 follow-up. Data were collected between November 2020 and January 2021, and the revision now demonstrates that infection-control rules directly shaped companion access, which is central to the paper's third theme. The limitations section, however, contains no mention of COVID-19. Please state plainly there that companion exclusion in particular is likely to reflect both structural features of Malawian obstetric care and pandemic-specific restrictions, and that the two cannot be fully separated with these data. Relatedly, the limitations section continues to claim a longitudinal perspective showing the evolution of practices, but the observation extracts are not dated or attributed to 2020/21 versus April 2024, so no temporal comparison is visible to the reader. Please either substantiate the claim by stating which patterns persisted in 2024 and labelling extracts by period, or soften it to what it actually is: a contextual re-immersion that supported interpretation.

2. Changes to Tables 1 and 2. Previously missing demographic data have been filled in. Public-hospital women's age range moves from "20-35 (NI: 3)" to "17-46" with the NI notation dropped, occupations are recoded from "Not Working (2), Business (3), Farming (2), NI (3)" to "Housewife (5), Business (3), Farming (2)", and the faith-based FGD row gains an age range and cadre description where it previously showed NI throughout. Parity still shows NI (3). Meanwhile the methods and limitations now state that demographic information was incomplete. Please explain in the methods how these data were subsequently obtained, and reconcile the tables with the new limitation statement. If the recoding of "Not Working" to "Housewife" reflects a reinterpretation rather than newly recovered data, please say so; the two terms are not equivalent and the change alters how the sample's variation reads.

3. Depth of the theoretical framework. The added classification, boundary and liminality language genuinely improves the results, and the softened abstract wording is appropriate. To finish the job, please align the methods with it. The methods still describe Douglas as providing the lens for theme formation and retain the rites-of-passage paragraph, while the results operationalize classification and boundary maintenance but not purity or pollution. Either state explicitly in the methods that the framework informed rather than structured the analysis, or add one or two sentences making the purity dimension analytically visible, for example in how the latent room separates the not-yet-clinically-legitimate from the admitted. Note also that Table 4's overarching theme, "The Imposition of Standardized Obstetric Admissions and Triage", was not updated when the three sub-themes were reworded and now reads in a different register from them.

4. Reporting items in the methods body. Interview duration, whether transcripts were returned to participants, and whether any member checking took place are still absent from the manuscript text. The SRQR checklist is welcome, but these items should also appear in the methods. One or two sentences will cover them.

5. Internal contradiction on companion access. The new study setting paragraph states that companions were permitted in the faith-based hospital's private birthing rooms but not in the government hospital's shared labor ward. A sentence retained later still reads that companions "were being introduced at the public hospital following improvements in labor ward privacy". Please reconcile these; the second sentence appears superseded. Since a companion focus group was conducted at the public hospital, it would also help to clarify that companions accompanied women to that facility even where they could not enter the ward.

6. Patient and public involvement. The new sentence acknowledging the absence of PPI as a constraint remains inside the standalone PPI section. Please relocate or duplicate it into the limitations section.

7. Language and citation numbering. PLOS does not copyedit, so please correct the following:

• Abstract: "little is know about how..." - this also appears in the abstract entered into the submission system, which is what is typeset, so it needs correcting in both places.

• Reflexivity: "The research team included also included both clinical and non-clinical researchers."

• Analysis section: "a theoretical lens to examine risk, danger within this space" - missing conjunction.

• Discussion: "systems must also recognize forms knowledge that are not explicitly clinical" - missing word.

• Introduction: "many low-and middle income countries" - hyphenation.

• Results: one residual present tense, "the admissions process becomes largely interrogative".

• Introduction, line 96: the citation (16) should be (17). The sentence on inadequate triage, equipment shortages, blood supply and overburdened staff draws on Mgawadere et al., now reference 17; reference 16 is the Rashidi Fakari narrative review on triage scales.

• Limitations, line 612: the citation (28-29) should be (29-30) for Malterud and Palinkas, consistent with the methods.

With these points addressed, the manuscript will make a useful contribution to the literature on how obstetric admission practices function where formal triage guidance is absent.

**********

what does this mean?). If published, this will include your full peer review and any attached files.

**Do you want your identity to be public for this peer review?**  If you choose “no”, your identity will remain anonymous but your review may still be made public.

For information about this choice, including consent withdrawal, please see our Privacy Policy

Reviewer #1: **Yes:**  Emilienne Carine Bieme-Ndi

**********

[NOTE: If reviewer comments were submitted as an attachment file, they will be attached to this email and accessible via the submission site. Please log into your account, locate the manuscript record, and check for the action link "View Attachments". If this link does not appear, there are no attachment files.]

**Figure Resubmissions:**

# Author comment: Author response to Decision Letter 2

**DOI:** 10.1371/journal.pgph.0006312.r004

22 Sep 2026

Attachment Submitted filename: Response_to_Reviewers_auresp_2.docx

# Peer review: Decision Letter 2

**DOI:** 10.1371/journal.pgph.0006312.r005

**Authors:** Julia Robinson

12 Oct 2026

Creating Order in the Uncertainty of Childbirth: A Qualitative Study of Obstetric Admission and Triage Practices in Rural Malawi

PGPH-D-26-01103R2

Dear Ms. Bundy,

We are pleased to inform you that your manuscript 'Creating Order in the Uncertainty of Childbirth: A Qualitative Study of Obstetric Admission and Triage Practices in Rural Malawi' has been provisionally accepted for publication in PLOS Global Public Health.

Before your manuscript can be formally accepted you will need to complete some formatting changes, which you will receive in a follow up email. A member of our team will be in touch with a set of requests.

Please note that your manuscript will not be scheduled for publication until you have made the required changes, so a swift response is appreciated.

IMPORTANT: The editorial review process is now complete. PLOS will only permit corrections to spelling, formatting or significant scientific errors from this point onwards. Requests for major changes, or any which affect the scientific understanding of your work, will cause delays to the publication date of your manuscript.

If your institution or institutions have a press office, please notify them about your upcoming paper to help maximize its impact. If they'll be preparing press materials, please inform our press team as soon as possible -- no later than 48 hours after receiving the formal acceptance. Your manuscript will remain under strict press embargo until 2 pm Eastern Time on the date of publication. For more information, please contact globalpubhealth@plos.org.

Thank you again for supporting Open Access publishing; we are looking forward to publishing your work in PLOS Global Public Health.

Best regards,

Julia Robinson

Executive Editor

PLOS Global Public Health

***********************************************************

Reviewer Comments (if any, and for reference):

Reviewer's Responses to Questions

**Comments to the Author**

Reviewer #1: All comments have been addressed

**********

publication criteria?

Reviewer #1: Yes

**********

3. Has the statistical analysis been performed appropriately and rigorously?

Reviewer #1: N/A

**********

4. Have the authors made all data underlying the findings in their manuscript fully available (please refer to the Data Availability Statement at the start of the manuscript PDF file)?

The PLOS Data policy

Reviewer #1: Yes

**********

5. Is the manuscript presented in an intelligible fashion and written in standard English?

Reviewer #1: Yes

**********

Reviewer #1: The COVID-19 limitation is now stated plainly and in the right place, and the longitudinal claim has been appropriately softened to contextual re-immersion. The theoretical framework is now described as having informed rather than structured the analysis, and Table 4's overarching theme has been rewritten so that it reads in the same register as the three sub-themes. The statement that transcripts were not returned and no member checking was conducted is an honest and appropriate addition. The contradiction on companion access is resolved in a single clear sentence that also accounts for companions waiting outside the public hospital, and the PPI constraint now appears in the limitations. The tables are now internally consistent with the incomplete-demographics statement, since NI notations have been retained wherever data genuinely remain missing. All earlier wording and citation corrections are in place, and the reference list is complete and correctly numbered throughout.

**********

what does this mean?). If published, this will include your full peer review and any attached files.

**Do you want your identity to be public for this peer review?**  If you choose “no”, your identity will remain anonymous but your review may still be made public.

For information about this choice, including consent withdrawal, please see our Privacy Policy

Reviewer #1: No

**********
